# In Vivo Reprogramming of Tissue‐Derived Extracellular Vesicles for Treating Chronic Tissue Injury Through Metabolic Engineering

**DOI:** 10.1002/advs.202415556

**Published:** 2025-03-31

**Authors:** Meng Zhao, Shuyun Liu, Yizhuo Wang, Peng Lou, Ke Lv, Tian Wu, Lan Li, Qianyi Wu, Jiaying Zhu, Yanrong Lu, Meihua Wan, Jingping Liu

**Affiliations:** ^1^ Department of General Surgery and NHC Key Laboratory of Transplant Engineering and Immunology Frontiers Science Center for Disease‐related Molecular Network West China Hospital Sichuan University Chengdu 610041 China; ^2^ Department of Emergency Guizhou Provincial People's Hospital Guiyang 550002 China; ^3^ West China Center of Excellence for Pancreatitis Institute of Integrated Traditional Chinese and Western Medicine West China Hospital Sichuan University Chengdu 610041 China

**Keywords:** chronic kidney disease, extracellular vesicle, metabolic engineering, mitochondrial biogenesis, skeletal muscle

## Abstract

Extracellular vesicles (EVs) have emerged as promising therapeutics for regenerative medicine, but the efficacy of current exogenous EV‐based therapies for treating chronic tissue injury is still unsatisfactory. Exercise can affect skeletal muscle EV secretion and that this process regulates the systemic health‐promoting role of exercise, suggesting that fine‐tuning of endogenous tissue EV secretion may provide a new therapeutic avenue. Here, this work reports that in vivo reprogramming of EV secretion via metabolic engineering is a promising strategy for treating chronic diseases. Briefly, exercise enhanced mitochondrial metabolism and EV production in healthy skeletal muscles, and EVs from healthy skeletal muscles subjected to exercise or metabolic engineering (boosting mitochondrial biogenesis via AAV‐mediated muscle‐specific TFAM overexpression) exerted cellular protective effects in vitro. In injured skeletal muscles, in vivo metabolic engineering therapy could reprogram EV secretion patterns (reducing pathological EV compositions while increasing beneficial EV compositions) by regulating multiple EV biogenesis and cargo sorting pathways. Reprogrammed muscle‐derived EVs could reach major organs and tissues via the circulation and then simultaneously attenuated multiple‐tissue (e.g., muscle and kidney) injury in chronic kidney disease. This study highlights that in vivo reprogramming of tissue‐derived EVs via a metabolic engineering approach is a potential strategy for treating diverse chronic diseases.

## Graphical abstract

Inspired by exercise‐induced metabolic rewiring and EV secretion, this study shows that in vivo metabolic engineering strategy can reprogram EV secretion pattern and simultaneously attenuate multiple tissue injury in CKD status, highlighting that it is a potential strategy for treating diverse chronic diseases.

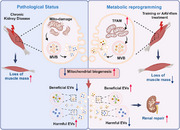

## 1 Introduction

To date, extracellular vesicle (EV)‐based nanotherapeutics have emerged as promising means for regenerative medicine in various forms of tissue injury.^[^ ^1^, ^2^, ^3^ ^]^ For example, we and others have reported that EVs from cultured stem cells (e.g., MSCs) or regulatory immune cells (e.g., M2 macrophages) can attenuate the inflammatory response and tissue injury in animal models of kidney injury and sepsis.^[^ ^3^, ^4^, ^5^, ^6^ ^]^ As cell‐derived natural nanovesicles, EVs present multiple advantages, such as high biosafety and biocompatibility, low immune reactivity, and the ability to cross biological barriers compared with synthetic nanoparticles.^[^ ^2^ ^]^ However, the clinical translation of current exogenous EV therapies is still unsatisfactory for several reasons. Currently, in vitro cell culture‐based therapeutic EV production has a relatively low yield, high cost and time consumption because long‐term culture of large numbers of donor cells, repeated collection and operation of large volumes of culture medium, and massive supplies of reagents/materials are needed.^[^ ^7^ ^]^ Moreover, exogenous EV treatments via systemic administration have relatively short half‐life and rapid clearance in vivo, which largely limits their tissue repair potential.^[^ ^8^ ^]^ These limitations can be further aggravated in the case of chronic disease treatment, since long‐term and consecutive dosing of exogenous EVs is needed to achieve the desired therapeutic effects.^[^ ^9^ ^]^ Therefore, novel strategies for EV therapy for chronic tissue injury are desirable.

EV secretion is a conserved biological process in most types of tissue cells, and tissue‐secreted endogenous EVs play vital roles in mediating cell‒cell and organ‒organ communication in both physiological and pathological states by transferring various bioactive cargos (e.g., proteins, nucleic acids, and lipids) between cells.^[^ ^2^ ^]^ Increasing evidence has indicated that healthy tissue‐secreted EVs can function in the regulation of metabolic status, tissue homeostasis and injury repair,^[^ ^7^, ^10^, ^11^ ^]^ whereas injured tissue‐secreted EVs can induce multiple pathogenic effects (e.g., metabolic disorders and inflammation) in diverse disease conditions in vivo.^[^ ^1^ ^]^ These findings suggest that reasonably modulating endogenous EV secretion (reducing harmful EVs or increasing beneficial EVs) may hold the potential for tissue injury repair and that in situ production of therapeutic EVs via tissue engineering tools may resolve the limitations of current cell culture‐based EV therapies and improve the potential of EV therapy for many chronic diseases. Skeletal muscle is one of the largest tissues (≈40% of body weight) in the body and can regulate systemic metabolism and physiological processes via secretory effects (e.g., myokines and EVs).^[^ ^12^ ^]^ Skeletal muscle‐secreted EVs can reach other tissues via the circulation and play vital roles in regulating many biological processes (e.g., cell metabolism, differentiation and injury repair) of targeted tissues,^[^ ^13^, ^14^ ^]^ suggesting that skeletal muscles are ideal tissues for producing engineered EVs in vivo.

Notably, exercise is a robust means to reshape skeletal muscle functions,^[^ ^15^ ^]^ and it can also affect EV secretion and/or composition in skeletal muscles.^[^ ^16^, ^17^ ^]^ Healthy human participants or mice present elevated levels of circulating EVs and/or altered cargos after exercise,^[^ ^16^, ^17^ ^]^ and exercise‐induced EVs have been shown to play multiple beneficial roles, such as improving the energy metabolism of adipose tissue and reducing cell death in ischemic tissue injury.^[^ ^17^, ^18^ ^]^ On the basis of these findings, we speculated that mimicking exercise via an engineering strategy may reprogram EV secretion in injured tissues. According to previous reports, the beneficial effect of acute exercise is due mainly to metabolic rewiring of muscles, especially increased mitochondrial metabolism.^[^ ^19^, ^20^ ^]^ For example, acute exercise has been shown to induce mitochondrial biogenesis and thus improve mitochondrial DNA (mtDNA) levels and mitochondrial respiration in the skeletal muscles (e.g., soleus) of mice.^[^ ^20^ ^]^ Importantly, increasing mitochondrial biogenesis via genetic tools can also increase mitochondrial oxidative metabolism and angiogenesis in skeletal muscles.^[^ ^21^ ^]^ Thus, in situ metabolic engineering of muscle via increased mitochondrial biogenesis may partially mimic the beneficial role of exercise in EV secretion, but its detailed effects have not been evaluated.

In this study, we report that in situ reprogramming of EV secretion in injured tissue via metabolic engineering is a promising strategy for treating chronic tissue injury. In brief, high‐intensity exercise (HIE) increased mitochondrial biogenesis and EV yield in healthy skeletal muscles. In vitro, EVs from healthy muscles with HIE or metabolic engineering (boosting mitochondrial biogenesis via AAV‐mediated muscle TFAM overexpression, TFAM‐OE) can reduce muscle or renal cell injury. In vivo, increasing muscle mitochondrial biogenesis via TFAM‐OE can reprogram EV secretion, with altered contents in injured muscle tissues of chronic kidney disease (CKD) mice. In situ reprogrammed skeletal muscle‐derived EVs arrived at major organs and tissues via the circulation and thus simultaneously reduced multiple‐tissue (e.g., muscle and kidney) injury in the CKD state. This study highlights that in situ reprogramming of tissue‐derived EVs via metabolic engineering is a potent strategy for treating diverse chronic diseases.

## 2 Results and Discussion

### 2.1 High‐Intensity Exercise Induces Metabolic Rewiring and EV Secretion in Healthy Skeletal Muscle

**Figure 1:**
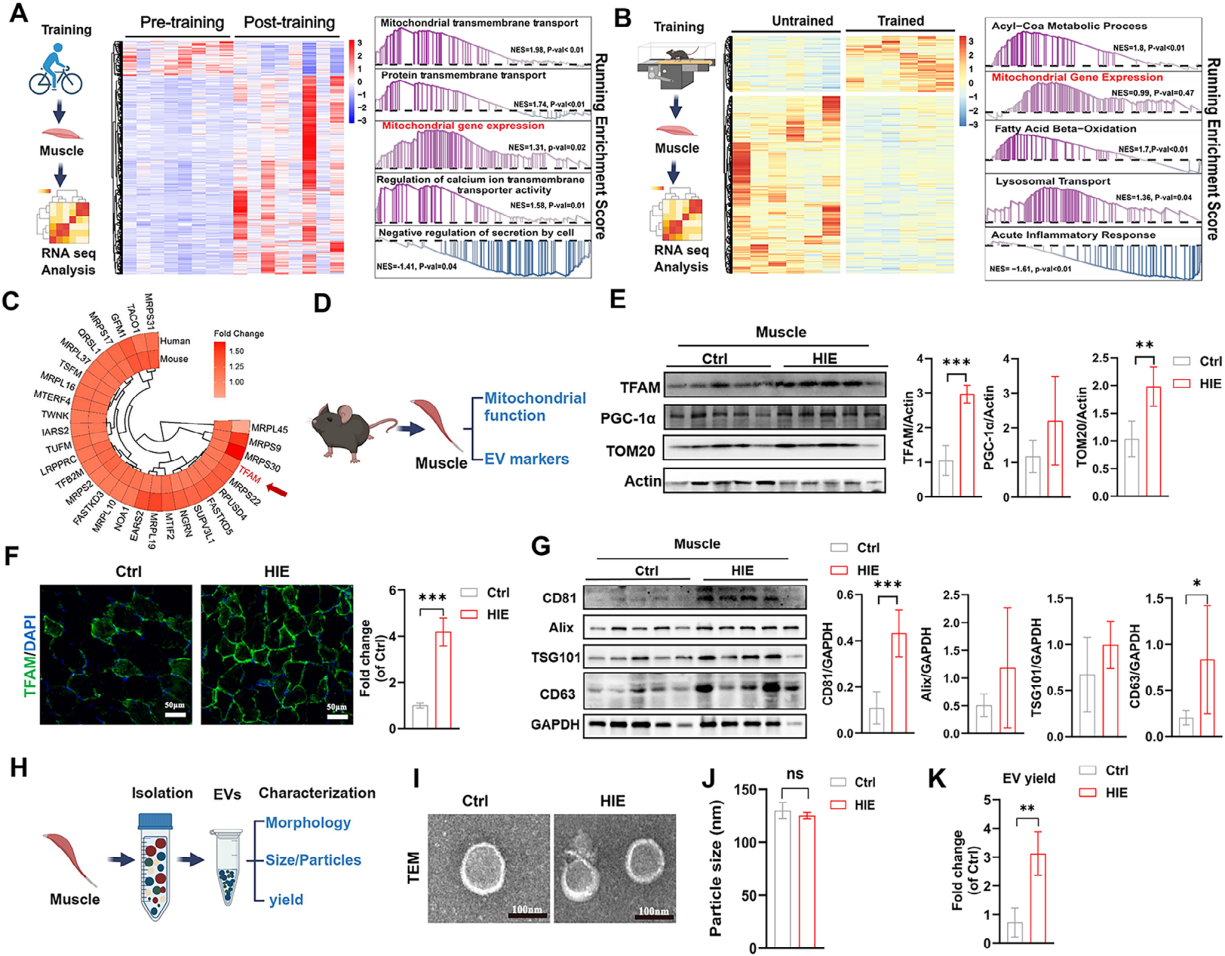
Exercise induces metabolic rewiring and EV secretion in healthy muscle tissue. A,B) Schematic processes of HIE and RNA‐seq analysis of human or mouse skeletal muscle from the GEO database (n = 8 subjects in A and n = 6 mice in B). C) Comparison of mitochondrial gene expression after HIE in humans and mice from the GEO database. D) Experimental scheme of HIE in this study. E) Western blot analysis and expression of mitochondrial proteins (TFAM, PGC‐1α, and TOM20) in skeletal muscle (n = 5 mice; **p < 0.01; ***p < 0.001). F) Representative images and quantification of TFAM in skeletal muscle after HIE (scale bar = 50 µm, n = 3 biological replicates, ***p < 0.001). G) Western blot analysis and expression of EV markers (CD81, Alix, CD63 and TSG101) in skeletal muscle (n = 5 mice; ***p < 0.001). H) Schematic processes of muscle‐EV production and characterization. I) Representative TEM images of EV preparations. J) Size distributions of EVs measured by NTA (n = 3 biological replicates). K) Comparison of EV yield (quantified by particle number and muscle weight) after HIE (n = 3 biological replicates, **p < 0.01).

Notably, exercise, such as HIE, has shown various benefits for the body across a broad age range, with improved physical functions, metabolic states, and quality of life.^[^ ^22^ ^]^ Interestingly, increasing evidence indicates that skeletal muscle‐secreted EVs might serve as vital intercellular communicators in mediating the systemic beneficial roles of exercise,^[^ ^23^ ^]^ but the possible factor of exercise in regulating EV secretion remains elusive. To explore global muscle gene expression in response to HIE, public RNA‐seq data from humans with long‐term HIE (1 h day^−1^, 5 days week^−1^ for 6 weeks; GSE144133) and mice with long‐term HIE (1 h day^−1^, 6 days week^−1^ for 8 weeks; GSE186251) were first analyzed. A heatmap revealed distinct gene expression patterns between the HIE (post‐training or trained) group and the control (pre‐training or untrained) group in both humans and mice (**Figure** **1**A,B). The differentially expressed genes (DEGs) between the HIE group and the control group in humans (533 upregulated, 94 downregulated) or mice (806 upregulated, 484 downregulated) were identified (Figure S1A,B, Supporting Information). Gene set enrichment analysis (GSEA) revealed that the upregulated genes associated with HIE in mice or humans were highly enriched in mitochondria‐related biological processes, such as increased mitochondrial gene expression, mitochondrial transmembrane transport and fatty acid β‐oxidation (Figure 1A,B). HIE intervention induced many shared mitochondrial DEGs (≈30) in the muscle of both human and mouse HIE, such as MRPLs, MRPSs, TFAM (mitochondrial transcription factor A), and TFB2 M (Figure 1C and Figure S1C, Supporting Information). HIE reportedly promotes mitochondrial biogenesis and mitochondrial oxidative phosphorylation (OXPHOS) in skeletal muscle.^[^ ^19^ ^]^ We further verified these findings in HIE mice (Figure 1D) and found that the muscle of the HIE group had higher levels of proteins related to mitochondrial biogenesis (e.g., TFAM and TOM20) and the mitochondrial ETC (e.g., NDUFB8, SDHB, UQCRC2, and ATP5a‐1) than the control group did (Figure 1E,F and Figure S2A,B, Supporting Information), suggesting that HIE may augment muscle function by enhancing mitochondrial biogenesis.

On the other hand, studies have shown that healthy muscle has increased EV release into the circulation in response to acute exercise in an intensity‐dependent manner.^[^ ^23^ ^]^ For example, exercise intervention induces the release of EVs with altered EV markers (e.g., ALIX, ACTN4, ADAM10, and CD81) from skeletal muscle into the circulation in healthy humans.^[^ ^24^ ^]^ Similarly, we found that the muscle tissues of normal mice with HIE expressed higher levels of vital EV biogenesis regulators, such as ALIX (involved in multivesicular body formation), TSG101 (involved in small ectosome formation), CD81 (involved in endosome formation), and CD63 (a membrane protein marker of small EVs (exosomes)), than those in the control group did (Figure 1G), suggesting that HIE can promote muscle EV secretion by affecting multiple biogenesis pathways. However, the increase in TSG101 and Alix expression did not reach significance compared with that in the control group (Figure 1G), possibly because individual pathways might respond differently to HIE stimulation or interact with other pathways (feedback effect), and the detailed reason needs to be revealed in future studies. Muscle EVs from control (Ctrl‐EVs) or HIE (HIE‐EVs) group mice were isolated and analyzed (Figure 1H). The isolated Ctrl‐EVs and HIE‐EVs were typical bilayer membrane vesicles (Figure 1I) with similar mean sizes (≈130 nm versus ≈125.22 nm) (Figure 1J and Figure S2C,D, Supporting Information), suggesting that HIE did not alter the microstructure or size distribution of muscle EVs. Moreover, the muscle EV yield (assessed by the EV/muscle mass ratio) of the HIE group was significantly greater (≈3.12‐fold) than that of the control group (Figure 1K). Together, these results suggest that HIE can promote EV secretion in healthy skeletal muscles and that this effect is likely due to increased mitochondrial biogenesis.

### 2.2 Metabolic Engineering Promotes the Secretion of Beneficial EVs in Healthy Skeletal Muscle

**Figure 2:**
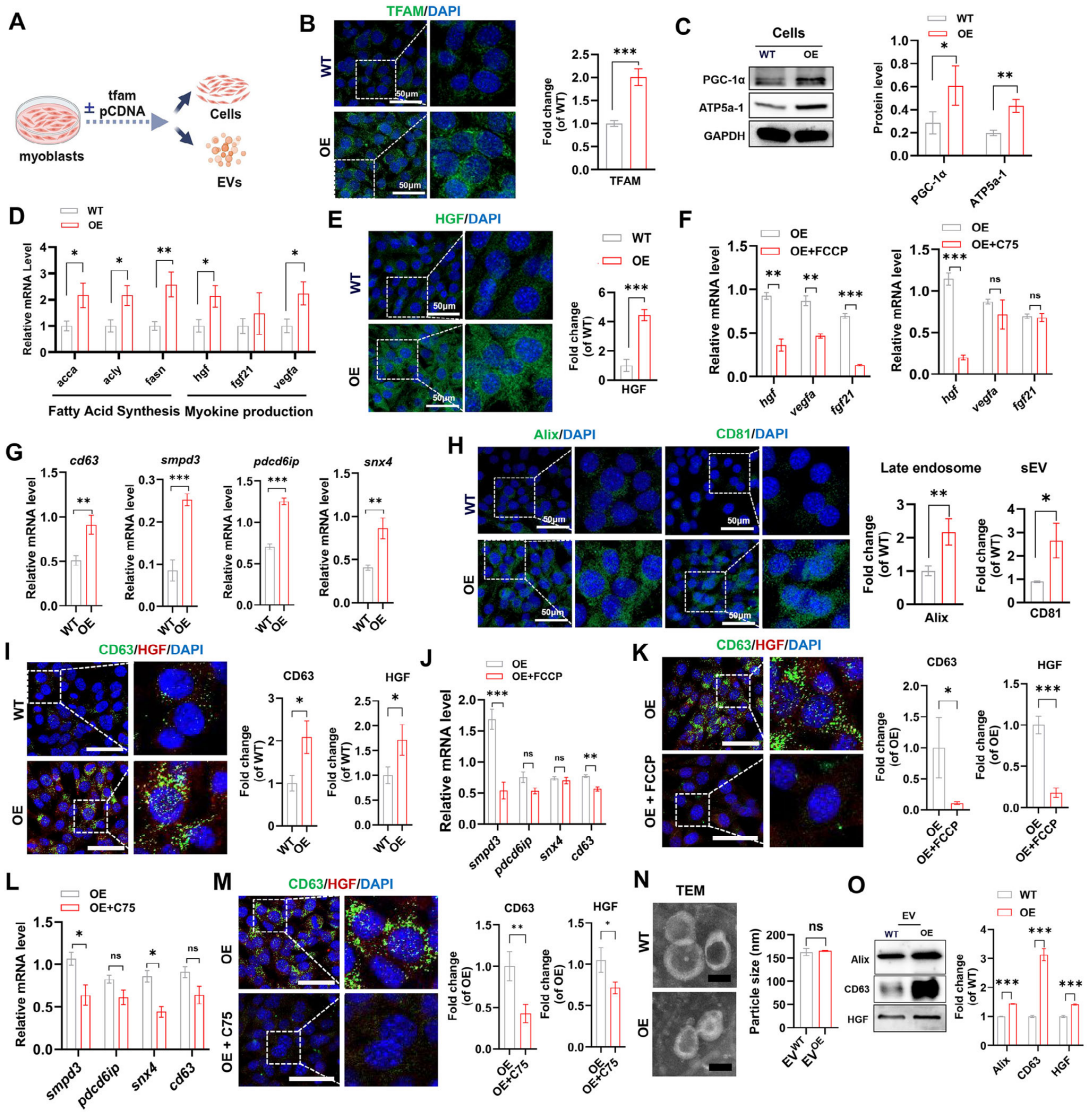
Metabolic engineering promotes the secretion of beneficial EVs in healthy skeletal muscle. A) Schematic diagram of the experimental process. Mouse myoblasts were transfected with normal control pcDNA (WT) or TFAM‐OE plasmid (OE), and the changes in the cells and EVs were analyzed. B) The relative protein levels of TFAM in myoblasts transfected with plasmid (scale bar = 50 µm; n = 3 biological replicates, ***p < 0.001). C) Western blot analysis and quantification of mitochondrial proteins (PGC‐1α and ATP5a‐1) in myoblasts transfected with plasmids for 24 h (n = 3 biological replicates, *p < 0.05, **p < 0.01). D) qPCR analysis of fatty acid synthesis‐related gene expression (acca, acly and fasn) and myokines (hgf, fgf21 and vegfa) in myoblasts after plasmid transfection for 24 h (n = 3 biological replicates, *p < 0.05, **p < 0.01). E) Representative micrographs and quantification of growth factor (HGF) IF staining in myoblasts after plasmid transfection for 24 h (scale bar = 50 µm, n = 3 biological replicates, ***p < 0.001). F) qPCR analysis of myokines (hgf, fgf21 and vegfa) in myoblasts after treatment with plasmid and FCCP or C75 for 24 h (n = 3 biological replicates, ^ns^ *p* > 0.05, **p < 0.01, ***p < 0.001). G) qPCR analysis of EV biogenesis‐related gene expression (cd63, smpd3, pdcd6ip and snx4) in myoblasts after plasmid transfection for 24 h (n = 3 biological replicates, **p < 0.01, ***p < 0.001). H) Representative micrographs and quantification of Alix and CD81 IF staining in myoblasts transfected with plasmid for 24 h (scale bar = 50 µm, n = 3 biological replicates, *p < 0.05, **p < 0.01). I) Representative micrographs and quantification of CD63 and HGF IF staining in myoblasts after plasmid transfection for 24 h (scale bar = 50 µm, n = 3 biological replicates, *p < 0.05). J) qPCR analysis of EV biogenesis‐related gene expression (cd63, smpd3, pdcd6ip and snx4) in myoblasts after treatment with plasmid and FCCP for 24 h (n = 3 biological replicates, ^ns^ *p* > 0.05, **p < 0.01, ***p < 0.001). K) Representative micrographs and quantification of CD63 and HGF IF staining in myoblasts after treatment with plasmid and FCCP for 24 h (scale bar = 50 µm, n = 3 biological replicates, ^ns^ *p* > 0.05, **p < 0.01, ***p < 0.001). L) qPCR analysis of EV biogenesis‐related gene expression (cd63, smpd3, pdcd6ip and snx4) in myoblasts after treatment with plasmid and C75 for 24 h (n = 3 biological replicates, ^ns^ *p* > 0.05, *p < 0.05). M) Representative micrographs and quantification of CD63 and HGF IF staining in myoblasts after treatment with plasmid and C75 for 24 h (scale bar = 50 µm, n = 3 biological replicates, *p < 0.05, **p < 0.01). N) Representative TEM images of EV preparations and size distributions of EVs measured by NTA (scale bar = 100 nm, n = 3 biological replicates, ^ns^ *p* > 0.05). O) Western blot and quantification analysis of EV markers (Alix and CD63) and HGF in EVs (n = 3 biological replicates, ***p < 0.001).

Next, we sought to assess whether metabolic engineering intervention via increased mitochondrial biogenesis can partially mimic the beneficial role of exercise in healthy skeletal muscles. As a vital mitochondrial biogenesis regulator, TFAM can promote the transcription and replication of the mitochondrial genome (mtDNA), leading to increased mtDNA copy number and the transcription of its encoded mitochondrial proteins, thus improving mitochondrial biogenesis, OXPHOS and energy production in skeletal muscle.^[^ ^25^ ^]^ After the upregulation of TFAM in the muscles of the HIE group (Figure 1C,E,F), we evaluated the impact of TFAM overexpression (TFAM‐OE) on the cell phenotype and EV secretion in mouse skeletal myoblasts (C2C12) via plasmid transfection (**Figure** **2**A). Compared with WT myoblasts, TFAM‐OE myoblasts presented higher levels of TFAM mRNA and protein (transfected with the blank plasmid) (Figure 2B and Figure S5A, Supporting Information) and elevated levels of the mitochondrial biogenesis mediator (PGC‐1α) and ETC protein (ATP5a‐1) (Figure 2C), confirming enhanced mitochondrial biogenesis in myoblasts via TFAM‐OE. Exercise can exert health‐promoting effects via the induction of metabolic alterations and the production of beneficial proteins (myokines), such as growth factors (GFs, e.g., VEGF, IGF1, and HGF), in muscles.^[^ ^26^ ^]^ Additionally, increased mitochondrial OXPHOS and associated fatty acid synthesis can increase GF (e.g., VEGF, HGF) production in stem cells.^[^ ^27^ ^]^ Similarly, normal myoblasts with TFAM‐OE presented elevated levels of fatty acid synthesis (e.g., acca, acly, and fasn) and GF production (e.g., hgf, fgf21, and vegfa) (Figure 2A–E), whereas this increase was partially abolished by disrupting mitochondrial oxidative phosphorylation (FCCP) or lipid synthesis (C75, fatty acid synthase inhibitor) (Figure 2F), confirming that these beneficial effects of TFAM‐OE are dependent on metabolic rewiring of myoblasts.

Notably, cellular EV secretion can be affected by metabolic states, such as mitochondrial metabolism^[^ ^28^ ^]^ and lipid metabolism, since lipid contents are essential components of EVs, with important structural and regulatory functions during EV biogenesis.^[^ ^29^ ^]^ EV are a group of heterogeneous lipid bilayer vesicles containing various subtypes (e.g., exosomes, ectosomes, and apoptotic vesicles), and diverse biogenesis mechanisms, such as endosomal sorting complexes required for transport (ESCRT)‐dependent pathways, ESCRT‐independent (e.g., lipid or tetraspanin‐driven) pathways, and ectosome blebbing, have been reported.^[^ ^30^ ^]^ The process of EV cargo sorting is also driven through recognition by the biogenesis machinery and by cargo‐specific interactions (active process) or increases in local cargo concentrations (passive process). To explore the possible mechanisms underlying TFAM‐OE‐induced EV secretion, the expression of key regulators related to different EV biogenesis and cargo sorting processes, such as ESCRT‐dependent (e.g., pdcd6ip, encoding Alix) and ESCRT‐independent pathways via lipids (smpd3, encoding neutral sphingomyelinases, nSMases) or tetraspanins (e.g., CD63), and the formation of recycling endosomes to the plasma membrane (e.g., snx4, sorting nexin 4)^[^ ^31^ ^]^ were further detected. As shown in Figure 2G, compared with control (WT) myoblasts, TFAM‐OE (OE) myoblasts presented higher levels of smpd3, pdcd6ip, cd63, and snx4. In contrast, the expression of these upregulated genes (smpd3, pdcd6ip, cd63, and snx4) could be reduced to some extent by disrupting mitochondrial (FCCP) or lipid metabolism (C75) (Figure 2J–L). These results suggest that TFAM‐OE might simultaneously upregulate multiple EV biogenesis and cargo sorting routes, including ESCRT‐dependent and ESCRT‐independent pathways, in myoblasts.

The formation of different types of endosomes and EV release consists of complicated and dynamic processes. For example, small EVs (exosomes) are formed in addition to the endosomal pathway and are initiated by the formation of intraluminal vesicles (ILVs) within multivesicular bodies (MVBs). ILVs are subsequently released as EVs into the extracellular space via the fusion of MVBs with the plasma membrane.^[^ ^32^ ^]^ Similarly, we also found that compared with those in the WT group, the number of EEA1^+^ early endosomes (Figure S3, Supporting Information), Alix^+^ late endosomes (Figure 2H), and CD63^+^ EVs (Figure 2I) in the myoblasts in the OE group was increased, whereas the formation of such endosomal compartments and EVs was reduced by FCCP (Figure 2K) or C75 (Figure 2M) treatment, suggesting that the dynamic processes of EV formation can be induced by TFAM‐OE‐mediated metabolic rewiring. Furthermore, increased levels of cargos (e.g., HGF) could be observed in EEA1^+^ early endosomes (Figure S3A, Supporting Information), CD63^+^ EVs (Figure 2I) and released EVs (Figure 2O), indicating that the sorting of beneficial cargos into EVs is a dynamic and complicated process. The myoblasts of the OE group presented slightly increased EV yields, whereas the sizes of the EVs were not affected (Figure 2N and Figure S5B,C, Supporting Information). In line with a previous report,^[^ ^5^ ^]^ EVs from myoblasts in the OE group presented higher levels of GFs (e.g., HGF) than those in the control group did, confirming that TFAM‐OE can promote the synthesis and sorting of beneficial cargos (e.g., GFs) into EVs in myoblasts. Together, these results suggest that muscle cells might simultaneously upregulate multiple EV biogenesis and cargo sorting pathways in response to metabolic rewiring.

To further explore the potential EV secretion patterns in muscles subjected to exercise or metabolic engineering in vivo, the composition signature of muscle tissue‐derived EVs from healthy mice subjected to exercise (EV^HIE^) or metabolic engineering (AAV‐mediated TFAM‐OE, EV^OE^) was assayed via proteomics analysis because there is no appropriate cell model that can exactly mimic exercise (HIE) in vitro, and EV cargo “fingerprints” can provide insights into the donor cell status and the potential pathways underlying EV secretion and cargo sorting.^[^ ^30^ ^]^ As shown in Figure S4, Supporting Information, there were many (more than ≈50%) shared proteins between the EV^OE^ group and the EV^HIE^ group (Figure S4D, Supporting Information), and these shared proteins were enriched in multiple pathways related to cell metabolism (e.g., mitochondrial organization, mitochondrial translation, amino acid metabolic processes, and lipid metabolic processes) and EV biogenesis (e.g., multivesicular body sorting, regulation of vesicle‐mediated transport, vesicle organization, and vesicle budding from the membrane), and the EV^OE^ group presented more abundant proteins related to mitochondrial metabolism, such as ATP metabolic processes, oxidative phosphorylation and the respiratory electron transport chain (Figure S4E,F, Supporting Information). These results suggest that both HIE and OE induce metabolic rewiring and EV biogenesis pathways in muscles and that OE may have a profound effect on mitochondrial metabolism. Further analysis revealed that both EV^OE^ and EV^HIE^ contained large numbers of proteins related to mitochondrial oxidative phosphorylation (e.g., Cox14, Ndufa2), lipid metabolism (e.g., Cpt1b, Cyp27a1), and ATP metabolic processes (e.g., Sdhb, Atp5pb, Ndufa7) (Figure S4G, Supporting Information), and the abundance of some mitochondrial proteins were greater in the EV^OE^ group (Figure S4G–H, Supporting Information). In addition, EV^OE^ and EV^HIE^ expressed many proteins involved in diverse pathways related to EV biogenesis and cargo sorting, such as vesicle location (e.g., Exoc7, Rab3a, Vps33a), ESCRT (e.g., Tsg101, Chnmp3, Vps37c) and vesicle budding (e.g., Trim72, Mia3, Vapb). Similar to our in vitro findings, these results verify that increased EV secretion by muscle cells under exercise or metabolic engineering conditions is jointly regulated by multiple EV biogenesis and cargo sorting pathways.

**Figure 3:**
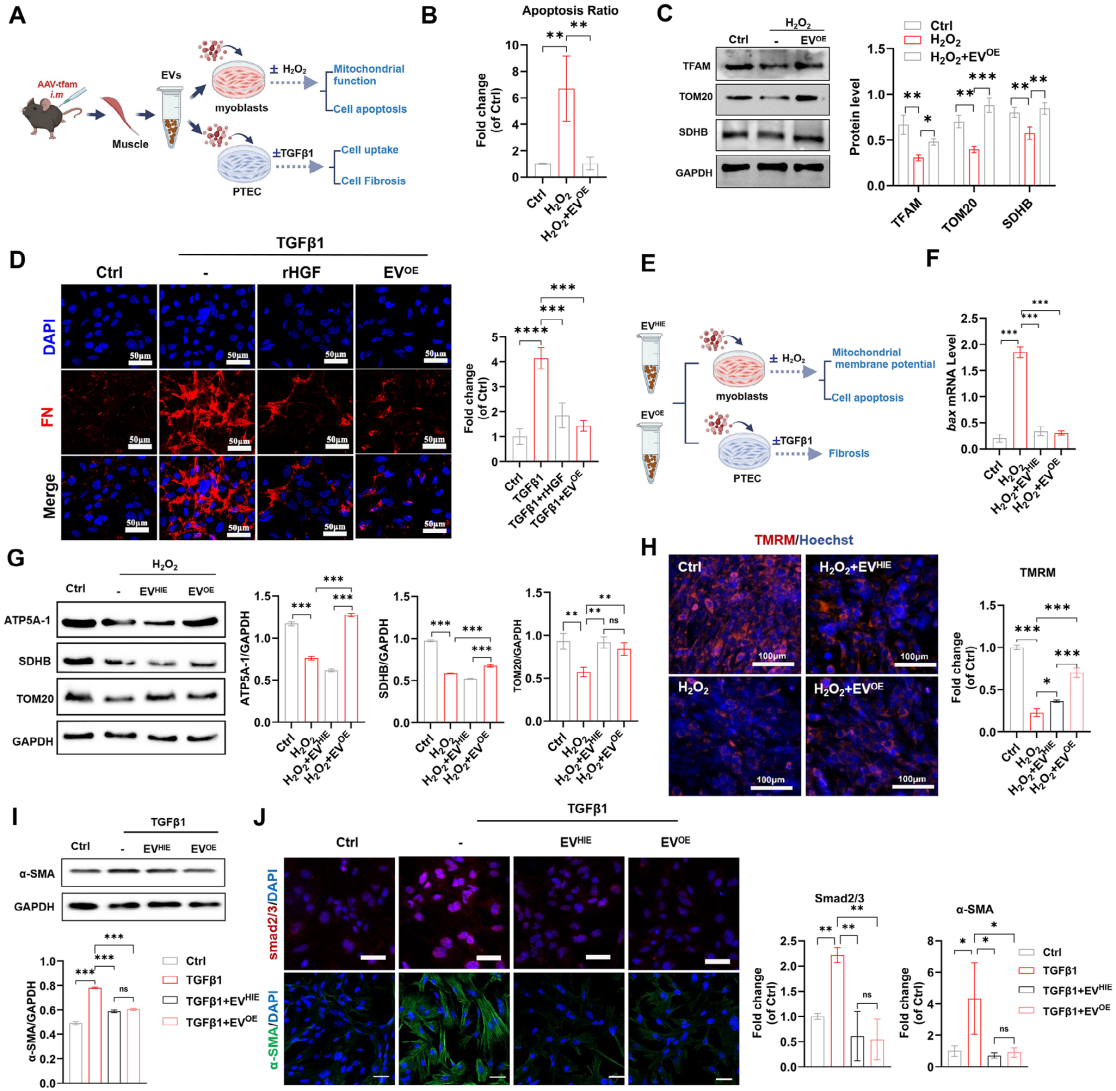
Muscle EVs from exercise or metabolic engineering conditions decrease muscle and kidney injury in vitro. A) Schematic illustrating the effect of muscle‐derived EVs on cells induced by H_2_O_2_ or TGFβ1. B) The ratio of apoptotic cells was determined via flow cytometry with Annexin V/PI staining. Damaged myoblasts (induced by 0.4 mM H_2_O_2_) were treated with EV^OE^ (≈6 × 10^9^ particles mL^−1^) for 48 h (n = 3 biological replicates; **p < 0.01). C) Western blot and quantification of the proteins (TFAM, TOM20 and SDHB) in myoblasts treated with EV^OE^ (≈6 ×10^9^ particles mL^−1^) under H_2_O_2_ (0.4 mM) stimulation for 48 h (n = 3 biological replicates; *p < 0.05; **p < 0.01; ***p < 0.001). D) Representative micrographs and quantification of FN IF staining in PTECs. PTECs were treated with rHGF (50 ng mL^−1^) or EV^OE^ (≈6 ×10^9^ particles mL^−1^) under TGFβ1 (10 ng mL^−1^) stimulation for 48 h (scale bar = 50 µm; n = 3 biological replicates; ***p < 0.001). E) Schematic illustrating the effects of muscle‐derived EVs from exercise (HIE) or metabolic engineering (OE) on cells induced by H_2_O_2_ or TGFβ1. F) qPCR analysis of bax gene expression in myoblasts treated with EV^HIE^ or EV^OE^ (≈6 ×10^9^ particles mL^−1^) under H_2_O_2_ (0.4 mM) stimulation for 48 h (n = 3 biological replicates; *** *p* < 0.001). G) Western blot analysis and quantification of mitochondrial proteins (ATP5a‐1, SDHB and TOM20) in myoblasts treated with EV^HIE^ or EV^OE^ (≈6 ×10^9^ particles mL^−1^) under H_2_O_2_ (0.4 mM) stimulation for 48 h (n = 3 biological replicates, **p < 0.01, ***p < 0.001, ^ns^ *p* > 0.05). H) Representative micrographs and quantification of TMRM staining in myoblasts treated with EV^HIE^ or EV^OE^ (≈6 ×10^9^ particles mL^−1^) under H_2_O_2_ (0.4 mM) stimulation for 48 h (scale bar = 100 µm, n = 3 biological replicates, *p < 0.05, ***p < 0.001). I) Western blot analysis and quantification of α‐SMA protein levels in PTECs treated with EV^HIE^ or EV^OE^ (≈6 ×10^9^ particles mL^−1^) under TGFβ1 (10 ng mL^−1^) stimulation for 48 h (n = 3 biological replicates, ^ns^ *p* > 0.05 ***p < 0.001). J) Representative micrographs and quantification of smad2/3 and α‐SMA IF staining in PTECs treated with EV^HIE^ or EV^OE^ (≈6 ×10^9^ particles mL^−1^) under TGFβ1 (10 ng mL^−1^) stimulation for 48 h (scale bar = 50 µm, n = 3 biological replicates, ^ns^ *p* > 0.05, *p < 0.05, **p < 0.01).

To date, the AAV vector has emerged as a pivotal delivery tool in clinical gene therapy owing to its minimal pathogenicity and ability to enable long‐term gene expression in diverse tissues. We conducted adeno‐associated virus (AAV)‐mediated muscle‐specific TFAM overexpression (AAV‐*tfam*) in healthy mice, isolated EVs from healthy mouse muscle tissues with TFAM‐OE (EV^OE^) on day 14 after AAV injection, and further evaluated the tissue protective role of EV^OE^ via diverse types of cell injury models (**Figure** **3**A). It has been reported that oxidative damage and associated mitochondrial injury and the apoptosis of skeletal muscle cells are vital pathological features of CKD‐related muscle injury.^[^ ^33^ ^]^ We also found that oxidative stress induced mitochondrial damage and cell apoptosis in myoblasts, as indicated by lower levels of mitochondrial proteins (TFAM, TOM20, and SDHB) and higher cell apoptotic rates in the H_2_O_2_ group than in the control group (Figure 3B,C). Muscle‐derived EV^OE^ could be efficiently taken up by myoblasts (Figure S5D, Supporting Information). The degree of mitochondrial injury and the rate of apoptosis in injured myoblasts were reduced by EV^OE^ treatment (Figure 3B,C and Figure S6A, Supporting Information). EV^OE^ treatment also promoted the migration of normal myoblasts (Figure S6B, Supporting Information). The progression of kidney fibrosis (excessive renal ECM deposition) is a hallmark of CKD, and epithelial‒mesenchymal transition (EMT) of renal tubular epithelial cells (PTECs) plays a role in the pathology of this process. GFs, such as HGF, can reduce renal fibrosis in UUO mice by inhibiting the expression of TGFβ1 and its receptor.^[^ ^34^ ^]^ In a TGFβ1‐induced renal tubular cell EMT model, we also found that muscle‐derived EV^OE^ could be taken up by PTECs and then effectively suppressed TGFβ1‐induced ECM protein (fibronectin, FN) expression in these cells (Figure 3D). Together, these results suggest that metabolic engineering via TFAM‐OE can induce the secretion of beneficial EVs in healthy skeletal muscles.

Next, the therapeutic potency of muscle EVs derived from exercise (EV^HIE^) or in vivo metabolic engineering (EV^OE^) was also assessed in vitro (Figure 3E). BCL‐2–associated X apoptosis regulator (Bax) is one of the core regulators of the intrinsic pathway of apoptosis and can induce cell death by forming pores in the mitochondrial outer membrane.^[^ ^35^ ^]^ In H_2_O_2_‐induced injured myoblasts, both the EV^HIE^ and EV^OE^ treatments had comparable effects on reducing bax expression (Figure 3F), suggesting that they can inhibit proapoptotic pathways in injured muscle cells. Additionally, EV^HIE^ or EV^OE^ treatment reversed the decrease in mitochondrial mass (TOM20) to some extent in injured myoblasts, whereas EV^OE^ had a greater protective effect on the expression of the mitochondrial proteins ATP5a‐1, TOM20 and SDHB (Figure 3G). The mitochondrial membrane potential (ΔΨm) generated by proton pumps is an essential component of the process of mitochondrial oxidative phosphorylation. The decreased ΔΨm (indicated by TMRM staining) of injured myoblasts could be restored by EV^HIE^ or EV^OE^, and EV^OE^ improved the ΔΨm better than EV^HIE^ did (Figure 3H). Collectively, these results suggest that the EV^HIE^ and EV^OE^ treatments have similar effects on protecting myoblasts from oxidative stress‐induced mitochondrial damage and cell apoptosis.

In addition, the renoprotective effects of EV^HIE^ or EV^OE^ were also evaluated in a TGFβ1‐induced EMT model of PTECs. α‐Smooth muscle actin (α‐SMA) is a specific marker for activated myofibroblasts and has been proposed to be an essential effector of tissue fibrosis. As shown in Figure 3I,J, EV^HIE^ or EV^OE^ treatment reduced the protein level of α‐SMA in TGFβ1‐induced PTECs, but there was no significant difference in α‐SMA expression between the two groups. The TGFβ/Smad signaling pathway plays a vital role in regulating EMT and tissue fibrosis, and the nuclear translocation of Smad 2/3 mediated by TGF‐β signaling can increase the expression of profibrotic factors. Consistently, increased levels of Smad 2/3 signals were observed in the nuclei of TGFβ1‐induced PTECs, which could be decreased after EV^HIE^ or EV^OE^, and there were no differences between the two groups (Figure 3J), suggesting that these EVs can attenuate the EMT and renal fibrotic process in PTECs. Together, these results indicate that both EVs from exercise and those from in vivo metabolic engineering have similar therapeutic effects on reducing muscle and kidney injury in vitro.

### 2.3 In Vivo Metabolic Engineering Reduces Mitochondrial Damage and Alters the EV Secretion Pathway in Injured Muscles

**Figure 4:**
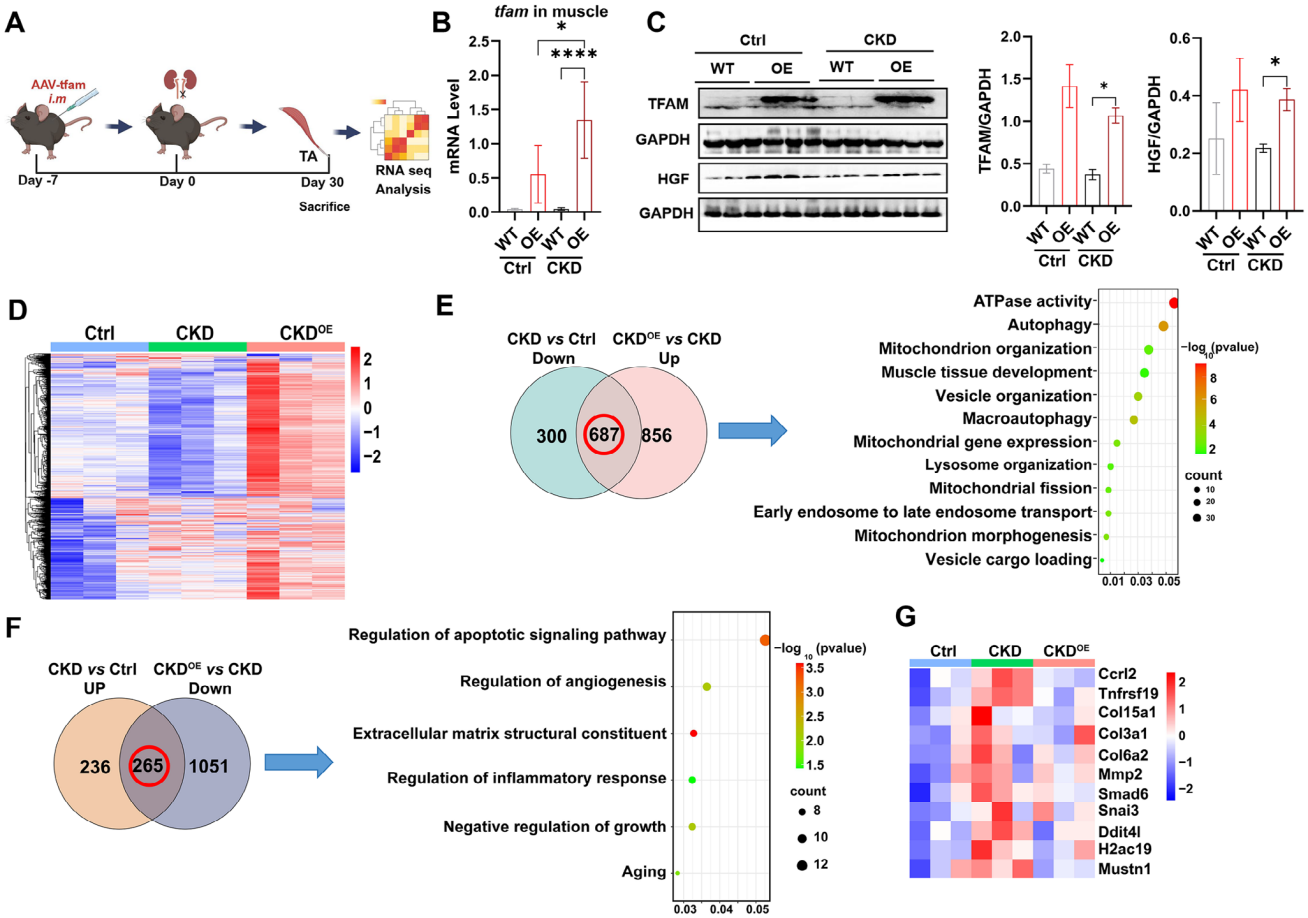
In vivo metabolic engineering alters the mitochondrial pathway in injured muscles. A) Schematic diagram of the experimental process. B) qPCR analysis of *tfam* expression in skeletal muscle (n = 6 mice; *p < 0.05, ****p < 0.0001). C) Western blot analysis and expression of mitochondrial proteins (TFAM) and growth factor (HGF) in skeletal muscle (n = 3 mice; *p < 0.05). D) Heatmap of the DEGs in skeletal muscle (n = 3 mice, FC > 1.2, *p* < 0.05). E) Venn diagram and GO enrichment analysis showing the DEGs downregulated between the CKD group and Ctrl group, and upregulated between the CKD^OE^ group and CKD group (FC > 1.2, *p* < 0.05). F) Venn diagram and GO enrichment analysis of DEGs upregulated between the CKD group and Ctrl group and downregulated between the CKD^OE^ group and CKD group (FC > 1.2, *p* < 0.05). G) Heatmap of the relative abundances of inflammation, fibrosis, and myokines in the three groups (n = 3 mice).

The impact of in vivo metabolic engineering on EV secretion was further assessed in injured tissues. However, as a proof‐of‐concept study, it is difficult to include too many disease models in a single study. In this study, CKD was used as a representative chronic disease model to evaluate the efficacy of our strategy because of its clinical significance and therapeutic challenge. CKD is a highly prevalent and serious chronic disease with high morbidity and mortality rates that can be caused by various insults, such as hypertension, diabetes, and autoimmune actions. It is estimated that CKD can affect ≈10% of the population worldwide and will become the fifth leading cause of death by 2050.^[^ ^36^ ^]^ Moreover, CKD is frequently associated with many other complications, such as chronic muscle injury (muscle atrophy), which causes a decrease in muscle function and further leads to an increased risk of morbidity and mortality in patients, but there is currently no effective therapy in the clinic.^[^ ^37^ ^]^ In this study, CKD mice presented chronic renal inflammation and fibrosis (excessive ECM deposition) (Figure S7, Supporting Information) and chronic skeletal muscle injury, as indicated by decreased body weight, grip strength, cross‐sectional area (CSA) of muscle fibers, disrupted CD31^+^ vessel networks and mitochondrial injury (Figures S8 and S9, Supporting Information). Interestingly, recent studies have revealed the beneficial effects of exercise on improving chronic inflammation, muscle function and metabolic states in CKD patients, but the underlying mechanism is still poorly understood.^[^ ^37^, ^38^ ^]^ The mice in the WT and OE groups were i.m. injected with muscle‐specific rAAV2/9‐CMV‐*tfam* vectors or AAV‐null vectors, respectively (**Figure** **4**A). TFAM overexpression (OE) at the gene and protein levels was confirmed in muscle tissues from normal control (Ctrl) and CKD mice (Figure 4B,C). In line with the cell test results, we also found that OE conditions upregulated GF (e.g., HGF) expression in the muscle of the Ctrl and CKD groups (Figure 4C), indicating that TFAM‐OE intervention can induce mitochondrial biogenesis and its downstream pathways.

The global changes in muscle gene expression with or without TFAM‐OE intervention were profiled via RNA‐seq (Figure 4D). The PCA plot and heatmap clearly revealed separation and distinct gene expression profiles among the control, CKD, and CKD plus tfam‐OE (CKD^OE^) groups (Figure 4D and Figure S10, Supporting Information). The DEGs between the groups were identified (Figure S10, Supporting Information). The pathological mechanism of CKD‐associated chronic muscle injury is complicated, and multiple factors, such as mitochondrial damage, inflammation, cell death, and fibrosis, are involved.^[^ ^39^ ^]^ GO enrichment analysis revealed that the impaired biological processes (downregulated DEGs, CKD versus control), such as mitochondrial energy metabolism, mitochondrial organization, mitochondrial fission, autophagy, muscle tissue development, vesicle organization, endosome transport, and vesicle cargo loading (Figure 4E), and the abnormally activated pathological processes (upregulated DEGs, CKD versus control), such as the apoptotic signaling pathway, ECM constituents, and the inflammatory response (Figure 4F), of CKD muscles could be reversed by TFAM‐OE intervention. In addition, the gene expression levels of proinflammatory cytokines (e.g., ccrl2, tnfsf19), ECM components (e.g., col3a1, col6a2, col15a1), profibrotic factors (e.g., mmp2, smad6, snail3), cellular metabolic stress responses (e.g., ddit4l), nucleosomes (e.g., h2ac19) and muscle injury (e.g., mustn1) in CKD muscle were attenuated by TFAM‐OE treatment (Figure 4G). These results collectively indicate that increasing mitochondrial biogenesis is sufficient to reduce mitochondrial injury and alter the EV secretion pattern in CKD muscle.

### 2.4 In Vivo Metabolic Engineering Reprograms the EV Secretion Pattern in Injured Muscle Tissue

**Figure 5:**
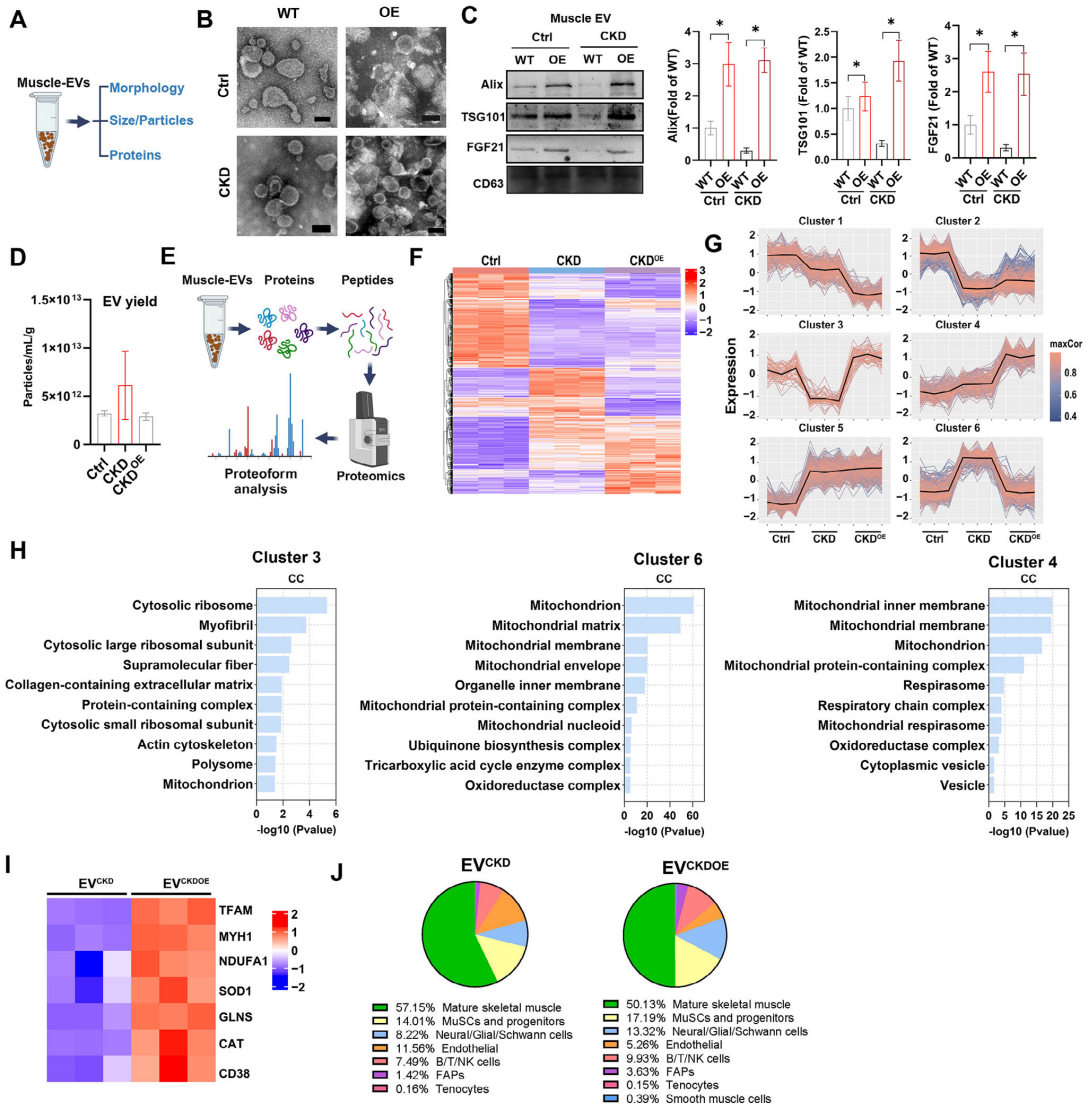
In vivo metabolic engineering reprograms EV secretion patterns in injured muscles. A) Schematic illustrating the evaluation of muscle‐EV production. B) Representative TEM images of EVs. C) Western blot analysis of EV proteins (Alix, TSG101, CD63 and FGF21) in equal amounts of EV samples with equal protein amounts (10 µg per panel; n = 3 biological replicates; *p < 0.05). D) EV yield analysis of muscle in mice after the administration of rAAV‐CMV‐*tfam* vectors (n = 3 biological replicates). E) Schematic illustrating the muscle‐EV proteomics experiments. F) Heatmap displaying variations in the protein content of muscle‐derived EVs in different groups (n = 3 mice, FC > 1.2 and p‐adjust < 0.05). G) Protein cluster trend plot of muscle‐derived EVs in different groups (n = 3 mice). H) GO enrichment analysis showing the DEPs of muscle EVs in each cluster. I) Heatmap of the relative abundance of mitochondrial proteins between the CKD^OE^ group and CKD group (n = 3 mice, FC > 1.2, and p‐adjust < 0.05). J) Percentage of EVs from each cluster relative to total EVs (tissue‐EV%).

To verify these findings, we isolated tissue EVs from mouse muscles via an ultracentrifugation‐based method as previously reported.^[^ ^40^ ^]^ The muscle EVs isolated from each group displayed a typical bilayer membrane vesicle structure, and their mean sizes were comparable (≈150 nm) (**Figures** **5**A,B and S11, Supporting Information); they expressed positive EV markers, such as Alix, TSG101 and CD63 (Figure 5C). Muscle EVs isolated via another method (Tim4 protein purification) also showed similar sizes between the WT groups and the OE groups in the control or CKD state (Figure S11D,E, Supporting Information). In line with the myoblast and muscle results, the muscle EVs of the OE group contained higher levels of GFs (e.g., FGF21) than those of the WT group did (Figure 5C). However, muscle tissues from CKD mice tended to have greater EV production (EV particle number/tissue weight) than did those from normal controls did, whereas this increase in EV production was reversed after TFAM‐OE (Figure 5D). Previous studies have shown that cells or tissues with oxidative stress or mitochondrial damage have increased release of EVs carrying damaged cell‐derived materials (e.g., injured mitochondria) in diverse forms of tissue injury (e.g., brain and adipose).^[^ ^41^ ^]^ Thus, the elevated muscle EV production during CKD is likely due to chronic muscle injury, while this effect can be attenuated by improving muscle mitochondrial function.

Notably, the diverse biofunctions of released EVs are largely dependent on their cargo signatures (e.g., proteins, nucleic acids).^[^ ^7^ ^]^ Given that increasing mitochondrial biogenesis affects muscle EV secretion, we next profiled the protein cargos of muscle EVs from the Ctrl, CKD (EV^CKD^) and CKD plus TFAM‐OE (EV^CKDOE^) groups via LC‒MS/MS‐based proteomics (Figure 5E). PCA scatter plots and heatmaps revealed distinct protein expression patterns between EV^Ctrl^, EV^CKD^ and EV^CKDOE^ (Figure 5F and Figure S12A–C, Supporting Information). The differentially expressed proteins (DEPs, p‐adjust value < 0.05) of EVs in the three groups were identified (Figure 5F). Further analysis revealed 6 different EV protein clusters on the basis of the DEGs between groups (Figure 5G). As shown in Figure 5H, some decreased EV proteins (versus the control) in the CKD state could be rescued by OE (cluster 3), and these proteins were enriched mainly in cellular components related to cytosolic ribosomes, myofibrils, and the actin cytoskeleton, suggesting that TFAM‐OE could rescue impaired protein synthesis and muscle function in CKD muscles. In contrast, some abnormally elevated EV proteins (versus the control) in the CKD state could be suppressed by OE (cluster 6), and they were enriched mainly in cellular components related to mitochondria, the mitochondrial matrix, the mitochondrial membrane and the ubiquinone biosynthesis complex, suggesting that TFAM‐OE could reduce mitochondrial injury and ubiquinone‐mediated excessive protein degradation in CKD muscles. Interestingly, some EV proteins were not affected by CKD but were increased by OE, and they were enriched in cellular components related to respiratory chain complexes and cytoplasmic vesicles, which might be due to altered EV biogenesis and cargo sorting processes.

Additionally, compared with EV^CKD^, EV^CKDOE^ carried more proteins related to mitochondrial function (e.g., TFAM and Ndufa1), glutamine synthesis (e.g., Glns), antioxidant enzymes (e.g., SOD1 and CAT), immune cell differentiation (e.g., CD38), and the number of slow myofibers (e.g., Myh1) (Figure 5I). These results suggest that increasing mitochondrial biogenesis can promote the production and sorting of multiple beneficial cargos into muscle EVs and thus contribute to the tissue protective effects of such EVs. Furthermore, the possible cell origins of EVs within muscle tissue were explored by mapping the EV proteomic data to public muscle single‐cell data as previously reported.^[^ ^42^ ^]^ The results revealed multiple major parent cell types of EV^CKD^ or EV^CKDOE^, such as mature skeletal muscle cells, MuSCs and progenitors, neural/glial/schwann cells and endothelial cells, in muscle tissues, and the EV^CKDOE^ group presented slightly increased populations of mature skeletal muscle cells, MuSCs and progenitors (Figure 5J). These results suggest that TFAM‐OE has a minor influence on the cell sources of EVs within muscle tissue. Together, our results suggest that increasing mitochondrial metabolism can reprogram EV secretion patterns (by reducing pathological cargo while increasing beneficial cargo) in injured muscle tissue.

### 2.5 In Vivo Reprogrammed Muscle‐Derived EVs can Reach Other Major Organs/Tissues via the Circulatory System

**Figure 6:**
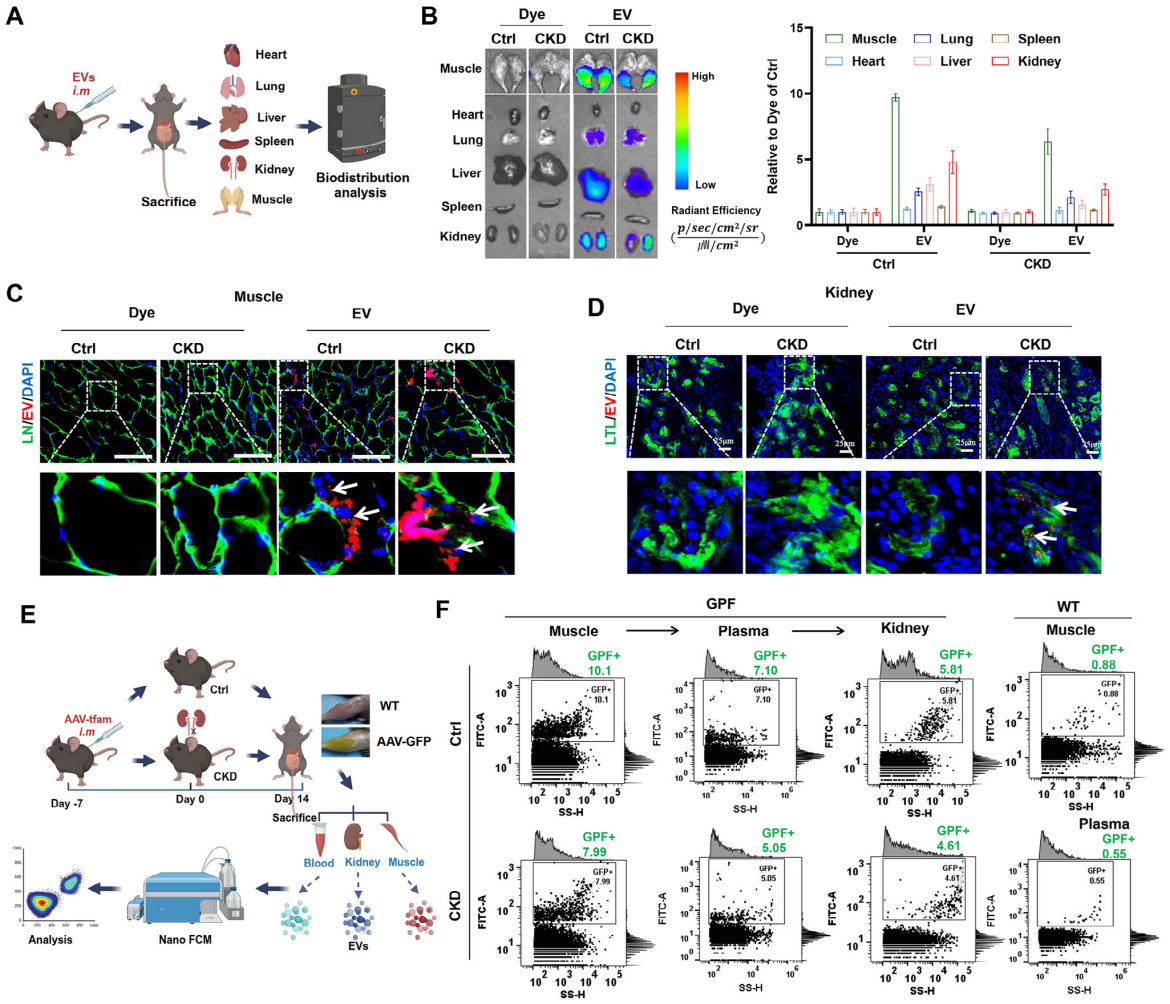
In vivo reprogrammed muscle‐derived EVs can reach other major organs and tissues via the circulatory system. A,B) Schematic illustration of EV biodistribution in different organs after muscle injection and representative IVIS images and quantification of organs and tissues (muscle, heart, lung, liver, spleen and kidney) harvested from mice at 4 h after Cy7‐EV (≈3.9 × 10^10^ particles mouse^−1^) injection. Mice that received Cy7 were used as negative controls (n = 3 mice). C) Representative micrographs of DiD‐EVs (red) in the muscle of mice at 4 h after DiD‐EV (≈3.9 × 10^10^ particles mouse^−1^) injection. The muscle was stained with LN (green), the cell nucleoli were stained with DAPI (blue), and the white arrows indicate EVs (scale bar = 100 µm). D) Representative micrographs of DiD‐EVs (red) in the kidneys of mice at 4 h after DiD‐EV (≈3.9 × 10^10^ particles mouse^−1^) injection. The renal tubules were stained with LTL (green), with DAPI (blue) indicating cell nucleoli and white arrows indicating EVs (scale bar = 100 µm). E,F) Schematic diagram of the experimental process: a029827 EVs were isolated from Ctrl or CKD mice (blood, muscle and kidney), which were subjected to specialized rAAV2/9‐ZsGreen vector administration, and the GFP‐positive signal of EVs was measured via Nano FCM.

In vivo, tissue cell‐derived EVs can impact the function of distal tissues or organs far from where they are secreted,^[^ ^5^, ^7^ ^]^ and muscle‐secreted EVs have been shown to enter the circulation and then reach multiple other organs, such as the liver, lung, kidney and spleen.^[^ ^43^, ^44^ ^]^ To assess the distribution of muscle‐derived EVs in vivo, myoblast‐derived EVs were labeled with Cy7 dye (Cy7‐EVs) and then intramuscularly (im) injected into the TA muscle of control or CKD mice (**Figure** **6**A). In addition to muscle tissues, Cy7‐EVs could be detected in other major organs, such as the liver, lung, kidney and spleen, at 4 or 24 h postinjection, especially the muscle and kidney, whereas no obvious signals were detected in any organ of the dye alone group (Figure 6B and Figure S13, Supporting Information). There was no significant difference in the EV signals of the tissues between the control and CKD groups at 24 h postinjection. A further validation test using another fluorescent dye (DiD) revealed a similar EV distribution pattern in vivo (Figure S14, Supporting Information), suggesting that muscle‐derived EVs can reach other organs.

To verify the results and exclude the possible bias in EV distribution caused by exogenous EV fusion, endogenous muscle‐secreted EVs were specifically labeled with a GFP tag. The TA muscles of the mice were i.m. injected with GFP‐overexpressing AAV (AAV‐GFP) or AAV‐null (WT), and these muscles strongly expressed GFP signals on day 21 post injection (Figure 6E). Then, EVs isolated from the blood, kidney, and muscle samples of the mice were analyzed via nanoFCM. In both normal and CKD mice, several GFP‐positive muscle EVs were detected in the muscle, blood, and kidney of the AAV‐GFP group, whereas a minimal signal was detected in the WT group (Figure 6F), which was in line with the results of dye‐labeled EVs. Furthermore, the distribution of muscle‐derived EVs within different tissues was examined. In vivo, signals from muscle‐derived EVs could be observed in the regions of LN‐labeled muscle fibers and LTL‐labeled renal tubular areas (Figure 6C,D). Together, these results indicate that muscle‐secreted EVs can enter other major organs via the circulation route.

### 2.6 In Vivo Reprogrammed Muscle EV Therapy Simultaneously Attenuates Multiple‐Tissue Injury in the CKD State

**Figure 7:**
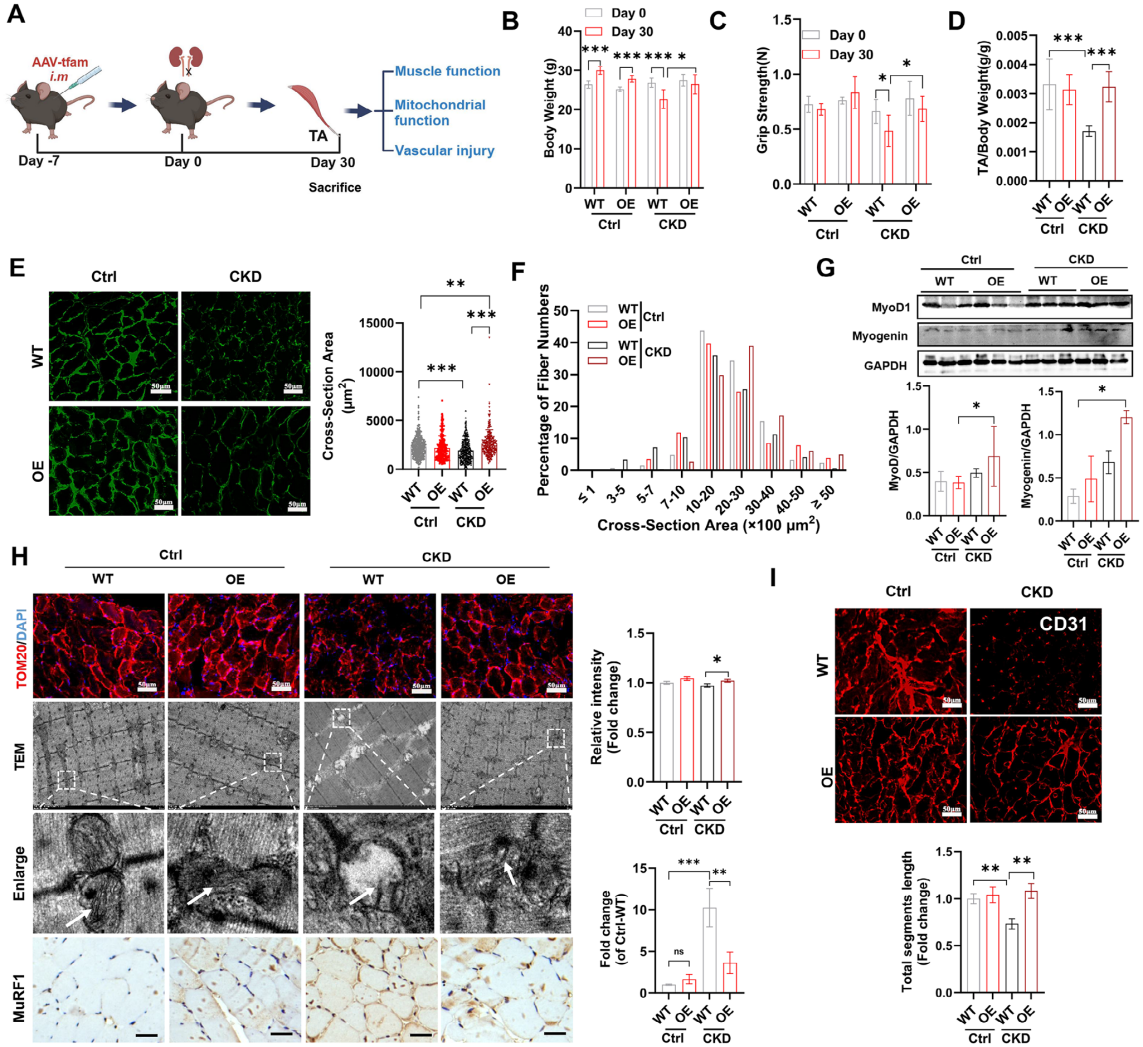
In vivo reprogrammed muscle EV therapy attenuates muscle and vascular injury in CKD mice. A) Schematic illustration of the animal experiment. B–D) Evaluation of muscle function (body weight, grip strength, and the ratio of tibialis anterior (TA) weight to body weight) in Ctrl or CKD mice on day 30 after the administration of AAV (n = 6 mice; *p < 0.05, **p < 0.01, ***p < 0.001). E) Representative micrographs of immunofluorescence analysis of LN expression in the TA and quantification of cross‐sections (n = 6 mice; **p < 0.01, ***p < 0.001). F) Bar graph showing the frequency distribution of fiber cross‐sectional areas in Ctrl or CKD mice on day 30 after the administration of AAV. G) Western blot and quantification of myokines (MyoD1 and myogenin) in the TAs of Ctrl or CKD mice on day 30 after the administration of rAAV (n = 3 mice; *p < 0.05). H) Representative micrographs of mitochondrial mass (indicated by TOM20, scale bar = 50 µm), morphology (observed via TEM, scale bar = 2 µm), and muscle injury marker (MuRF1) IHC staining (scale bar = 25 µm) in the TAs of CKD mice. The nuclei were stained with DAPI (blue) (n = 6 mice; *p < 0.05, **p < 0.01, ***p < 0.001). I) Representative structural micrographs and total segment length of vessels (indicated by CD31) in the TAs of Ctrl or CKD mice on day 30 after the administration of AAV (scale bar = 50 µm; n = 6 mice; **p < 0.01).

Because metabolic engineering via TFAM‐OE can induce functional EV secretion in muscle cells, we sought to evaluate whether this strategy can attenuate chronic tissue injury in a mouse CKD model (**Figure** **7**A). CKD is a high‐risk chronic metabolic disease with high morbidity and mortality rates and is accompanied by many complications (e.g., muscle atrophy) in addition to renal injury. Compared with those in the CKD alone group, the body weight, TA muscle weight, grip strength, and muscle cross‐sectional area of the mice in the CKD + OE group were greater (Figure 7B–E). Analysis of the fiber area frequency distribution revealed markedly elevated small fiber percentages but reduced larger fiber percentages (a leftward shift) in the muscles of CKD mice, while these effects could be reversed by OE intervention (Figure 7F). The pathology of CKD‐related skeletal muscle injury is complicated, and multiple factors, such as oxidative stress, metabolic disorders, vascular injury, and disturbed myogenesis, are involved.^[^ ^39^ ^]^ In the muscles of the CKD + OE group, the decreases in mitochondrial mass (TOM20), mitochondrial structure injury (e.g., ruptured mitochondrial membrane, irregular cristae), and vascular network integrity were markedly reversed after TFAM‐OE intervention (Figure 7H,I). Muscle is one of the most metabolically active tissues in the body, and its physiological functions depend on sufficient nutrients and oxygen transported via the microvascular network, while a decline in microvascular function is strongly associated with muscle atrophy. The CKD group presented decreased levels of muscle microvascular density and integrity (indicated by CD31 staining), which could be partially restored by TFAM‐OE treatment (Figure 7I), suggesting that the muscle‐protective role of TFAM‐OE is at least partly by restoring microvascular function.

In addition, myogenic regulatory factors, such as MyoD and myogenin, play crucial roles in directing satellite cell function to regenerate skeletal muscle.^[^ ^45^ ^]^ Interestingly, the muscles of the CKD + OE group presented increased MyoD and myogenin expression compared with those of the CKD alone group (Figure 7G). Muscle RING‐finger protein‐1 (MuRF1), an E3 ubiquitin ligase, plays a vital role in regulating muscle catabolism and remodeling. It has been reported that abnormal upregulation of MuRF1 expression is a critical factor of skeletal muscle atrophy.^[^ ^46^ ^]^ In line with a previous report, elevated levels of MuRF1 protein were found in the muscles of CKD mice, while MuRF1 expression could be partially suppressed by TFAM‐OE treatment (Figure 7H). Together, these data suggest that our in situ reprogrammed muscle EV therapy can improve muscle function in the CKD state by reducing mitochondrial damage and vascular injury while promoting myogenesis.

**Figure 8:**
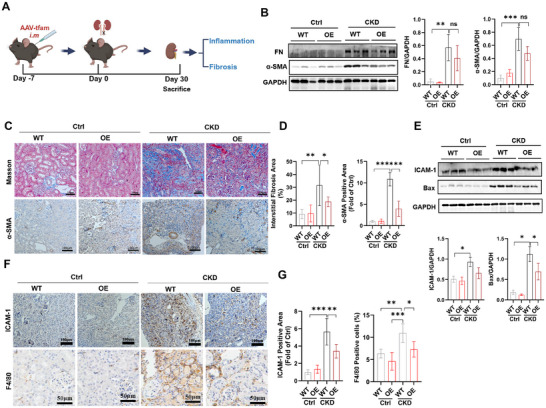
In vivo reprogrammed muscle EV therapy attenuates kidney injury in CKD conditions. A) Schematic illustration of the animal experiment. B) Western blot analysis and quantification of fibrosis‐related proteins (FN and α‐SMA) in the kidneys of mice on day 30 after the administration of AAV (n = 3 mice; **p < 0.01, ***p < 0.001). C‐D) Representative images and quantification of Masson staining and IHC staining (α‐SMA) of kidney tissues on day 30 after the administration of AAV (scale bar = 100 µm, n = 6 mice; *p < 0.05, **p < 0.01, ***p < 0.001). E) Western blot and quantification of the proteins (ICAM‐1 and Bax) in the kidneys of the mice on day 30 after the administration of AAV (n = 3 mice; *p < 0.05). F–G) Representative images and quantification of IHC staining (ICAM‐1 and F4/80) of kidney tissues on day 30 after the administration of AAV (scale bar = 100 µm for ICAM‐1 staining, and scale bar = 50 µm for F4/80 staining; n = 6 mice; *p < 0.05, **p < 0.01, ***p < 0.001).

**Figure 9:**
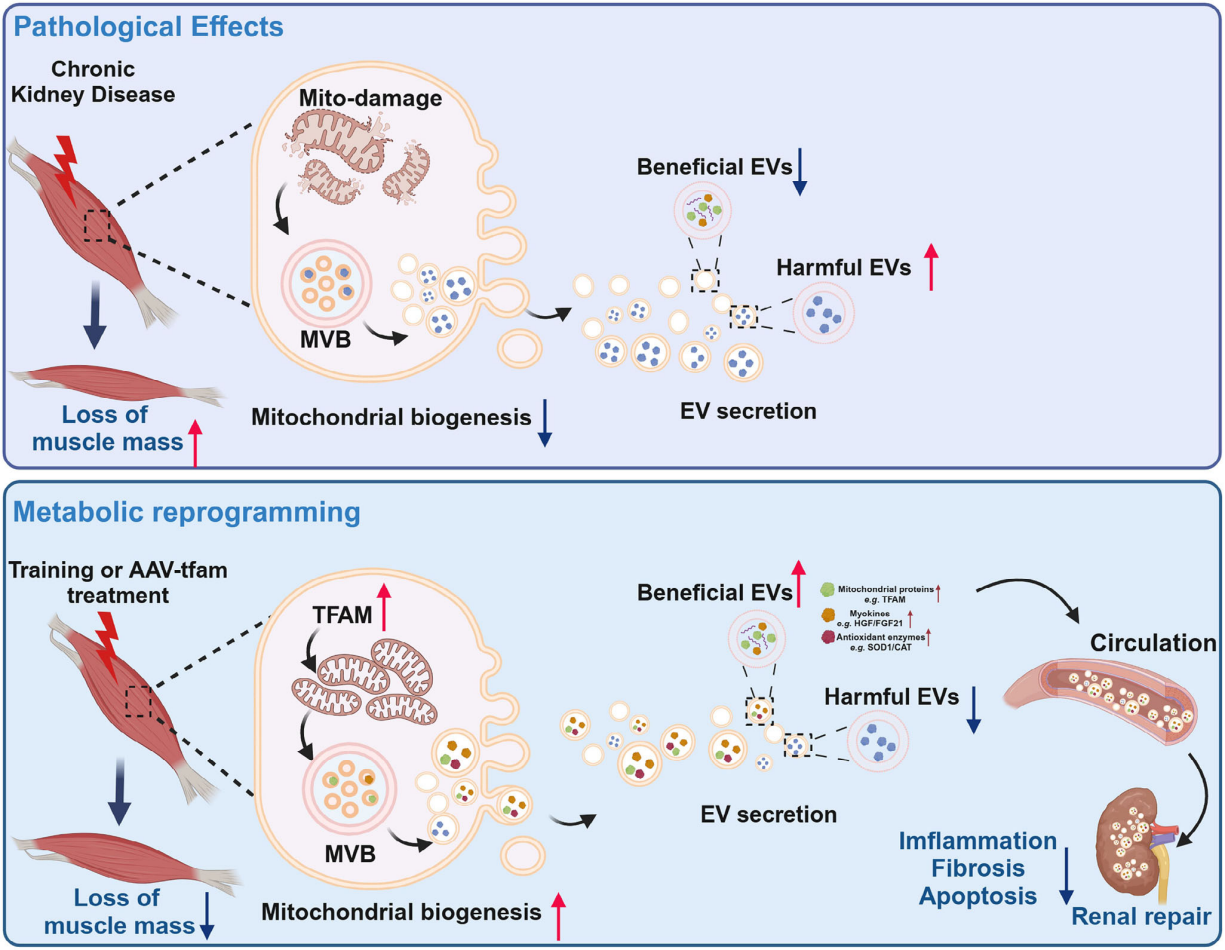
Schematic illustration of the key findings of this study. An in vivo metabolic engineering strategy can reprogram EV secretion patterns in injured skeletal muscles and simultaneously attenuate multiple‐tissue injury in CKD status.

Notably, chronic inflammation (increased proinflammatory cell infiltration and cytokine release) and kidney fibrosis (myofibroblast activation and excessive ECM deposition) are hallmark manifestations of progressive CKD.^[^ ^47^ ^]^ However, no efficient therapies against CKD currently exist in the clinic. Thus, we also assessed whether in situ reprogrammed muscle EV therapy can reduce distal organ injury far from muscles, and the changes in renal inflammation and fibrosis in CKD mice with or without muscle TFAM‐OE were analyzed (**Figure** **8**A). Compared with control mice, CKD mice presented elevated renal inflammation and fibrosis, as indicated by increased levels of F4/80^+^ macrophages and cytokine (e.g., ICAM‐1, TNF‐α, and IL‐6) expression, as well as α‐SMA^+^ activated myofibroblasts and interstitial ECM component (e.g., collagens and FN) deposition (Figure 8B–G, Figures S7 and S15B, Supporting Information). In contrast, the kidneys of mice subjected to muscle TFAM‐OE intervention presented lower levels of proinflammatory cell infiltration, cytokine expression, myofibroblasts and ECM deposition (Figure 8B–G). In addition, the increase in the protein expression of Bax (a vital mediator of cell apoptosis) in the kidneys of CKD mice was reversed by muscle TFAM‐OE intervention (Figure 8E), suggesting suppressed cell death in these kidneys. Moreover, muscle AAV‐TFAM injection did not increase TFAM expression in the kidneys of CKD mice (Figure S15A, Supporting Information), which excluded the direct rescue effect of AAV‐TFAM on renal injury. Together, our results indicate that in situ reprogrammed muscle EVs carrying diverse bioactive cargos can reach both nearby tissues (e.g., muscles) and distal organs (e.g., kidneys) to directly attenuate chronic tissue injury in the CKD state (**Figure** **9**).

To date, many clinical trials have reported the overall therapeutic benefit and safety of AAV vector‐mediated gene therapy in multiple diseases (e.g., neurological disorders), and it has been approved for clinical applications in the United States and Europe. Although the AAV‐TFAM vector was capable of enhancing muscle mitochondrial biogenesis and beneficial EV secretion in vivo, its long‐term biosafety and potential immunogenicity are unclear and need to be revealed. To this end, healthy mice were i.m. injected with the viral vector and monitored for up to 30 days. As shown in Figure S16C, Supporting Information, the pathological examination results revealed no obvious tissue lesions in the major organs, including the heart, liver, spleen, lung, kidney and muscle, of the AAV‐TFAM group on day 30 compared with those of the normal control group (WT). There was also no significant difference in the levels of renal function indicators (CREA and UREA) or liver function indicators (ALT and AST) between the WT group and the AAV‐TFAM group (Figure S16A,B, Supporting Information). Moreover, the effects of viral vectors on the innate immune response and the release of proinflammatory cytokines (e.g., IL‐6 and IL‐1β) were assessed in the blood samples of the mice, there was no significant difference in the levels of the proinflammatory cytokine (IL‐6) between the WT group and the AAV‐TFAM group (Figure S17A, Supporting Information). In multiple organs/tissues (including muscle, kidney, heart, liver, spleen and lung) of the mice, there was also no significant difference in the levels of cytokines (IL‐6 and IL‐1β) between the WT group and the AAV‐TFAM group (Figure S17B–G, Supporting Information). These results collectively indicate that this AAV‐TFAM vector has ideal biosafety and low immunogenicity in vivo.

Although the existing results are encouraging, some important questions have not been answered and need to be explored in future studies. For example, metabolic regulation by enhancing mitochondrial biogenesis can partially mimic the beneficial effect of exercise to reprogram EV secretion patterns in injured muscles, and such EVs have shown tissue protective effects in vitro and in vivo. However, fully excluding the possible contributions of other muscle‐released factors, such as soluble proteins and cytokines, to these processes is difficult. Muscle tissues consist of multiple cell types, thus, in addition to myoblasts, some other cell types may also contribute to beneficial EV secretion in skeletal muscles. Although we have shown that increased mitochondrial metabolism can affect pathways related to EV biogenesis and cargo sorting in muscle cells, the detailed underlying mechanism is unclear and needs to be revealed in future studies. Additionally, many other types of chronic diseases can also induce severe outcomes in patients and still lack efficient therapies, and the therapeutic potential of our strategy needs to be verified in other types of chronic disease models and large animal models. Despite previous reports and our results, the biosafety and low immunogenicity of the AAV vector in vivo might hold promise for future clinical translation. However, the potent adverse events associated with AAV gene therapy in vivo should not be overlooked, as reports from recent AAV clinical trials have shown that the risks of AAV sometimes overshadow the hopes for curing a hereditary disease.^[^ ^48^ ^]^ Thus, the long‐term safety and immunogenicity of AAV vectors need to be carefully assessed in additional model types. To overcome this limitation, alternative approaches, such as nonviral vectors and nanocarriers, may provide potential solutions to mitigate side effects. Nevertheless, this study highlights that metabolic states can regulate tissue EV secretion in vivo and that in situ reprogramming of tissue‐derived EVs via metabolic engineering is a potential strategy for treating diverse forms of chronic diseases.

## 3 Experimental Section

### Analysis of Muscle RNA‐seq Data with Exercise

Public muscle RNA‐seq data related to exercise, including human (GSE144133) and mouse (GSE186251) data, were obtained from the Gene Expression Omnibus (GEO) database. GSE144133 provides RNA‐seq data from human biopsies from musculus vastus muscle before and after acute supervised ergo cycle training, and GSE186251 provides RNA‐seq data from mouse skeletal muscle tissue with or without continuous high‐intensity exercise. The DEGs (FC > 1.5 and *p* value < 0.05) were identified via the DESeq2 R package (1.16.1). GSEA was implemented via the clusterProfiler package. ggplot2 (version 3.4.4) was used to visualize the analysis results, such as by generating PCA plots, heatmaps and volcano plots in the R script (version 4.2.3).

### Mouse Model of High‐Intensity Exercise

All animal experiments were approved by the Animal Care and Use Committee of West China Hospital, Sichuan University (Permit No. 20211551A) and followed the guidelines of the National Institutes of Health (NIH). C57BL/6 mice (male, 8–10 weeks old) were purchased from Byrness Weil Biotech Ltd. (Chengdu, China) and maintained in a standard environment in a pathogen‐free facility. Normal mice were subjected to a modified high‐intensity exercise (HIE) protocol as previously reported.^[^ ^49^ ^]^ Briefly, the mice were adapted to running on a treadmill for 3 days before exercise training. The HIE procedure included a warm‐up session (8 m min^−1^ for 8 min), followed by a training session consisting of two series of 8 min. Each series consisted of four high‐intensity sprints (16 m min^−1^, 8 min, 24 m min^−1^, 8 min) and low‐intensity walking speeds (13 m min^−1^, 8 min). A 5‐min passive rest (6 m min^−1^) was performed at the end of each series.

### Western Blotting Analysis

The cells, tissues, or EV samples were lysed in radioimmunoprecipitation assay (RIPA, P0013B, Beyotime Biotechnology, Shanghai, China) buffer supplemented with protease inhibitors (P1005, Beyotime Biotechnology) on ice. The protein concentration was determined with a BCA protein assay kit (CW0014S, CWBIO, Beijing, China). Protein expression was assayed via 10% or 12.5% sodium dodecyl sulfate‒polyacrylamide gel (SDS‒PAGE, PG112, PG113, Epizyme Biomedical Technology Co., Ltd., Shanghai, China) electrophoresis, after which the proteins were transferred to polyvinylidene difluoride (PVDF) membranes (ISEQ00010, Merck Millipore, Billerica, MA, USA). The membranes were blocked with 5% nonfat milk and incubated with primary antibodies against rabbit anti‐TFAM (1:500, 22586‐1‐AP, Proteintech, Wuhan, China), rabbit anti‐TOM20 (1:1000, 42406S, Cell Signaling Technology, Danvers, MA, USA), mouse anti‐PGC‐1α (1:500, 66369‐1‐Ig, Proteintech, Wuhan, China), rabbit anti‐NDUFB8 (1:500, A20457, ABclonal, Wuhan, China), rabbit anti‐SDHB (1:500, A1809, ABclonal, Wuhan, China), rabbit anti‐UQCRC2 (1:500, A4366, ABclonal, Wuhan, China), rabbit anti‐ATP5a‐1 (1:500, A5884, ABclonal, Wuhan, China), rabbit anti‐Bax (1:1000, #2772, Cell Signaling Technology, Danvers, MA, USA), anti‐ICAM‐1 (1:1000, 10831‐1‐AP, Proteintech, Wuhan, China), rabbit anti‐CD81 (1:1000, ab109201, Abcam, Cambridge, MA, USA), rabbit anti‐TSG101 (1:1000, 28283‐1‐AP, Proteintech, Wuhan, China), rabbit anti‐Alix (1:1000, 12422‐1‐AP, Proteintech, Wuhan, China), rabbit anti‐myogenin (1:500, A6664, ABclonal, Wuhan, China), rabbit anti‐CD63 (1:1000, ab217345, Abcam, Cambridge, MA, USA), rabbit anti‐α‐SMA (1:1000, ab124964, Abcam, Cambridge, MA, USA), rabbit anti‐HGF (1:1000, 26881‐1‐AP, Proteintech, Wuhan, China), rabbit anti‐FGF21 (1:1000, ab171941, Abcam,Cambridge, MA, USA), rabbit anti‐MyoD1 (1:500, 18943‐1‐AP, Proteintech,Wuhan, China), and rabbit anti‐Fibronectin (1:1000, FN, 15613‐1‐AP, Proteintech, Wuhan, China). After being washed with PBST, the PVDF membrane was incubated with the corresponding horseradish peroxidase‐conjugated secondary antibody (1:2000, ZB2301 or ZB2305; Zhongshanjinqiao Biotechnology, Beijing, China) at 37 °C for 1 h. An enhanced chemiluminescence kit (WBKLS, Merck Millipore) was used for signal detection. Protein bands were quantified via ImageJ software (NIH, Bethesda, MD, USA) and normalized to the expression of GAPDH (1:2000, A19056, ABclonal, Wuhan, China) or β‐actin (1:100 000, AC026, ABclonal, Wuhan, China).

### Immunofluorescence Staining

The slides or tissue sections were fixed with 4% paraformaldehyde in PBS for 10 min at room temperature and then permeabilized with 0.03% Triton X‐100 (T8787, Sigma‒Aldrich, MO, USA) for 10 min. After being blocked in 1% bovine serum albumin (BSA, 4240GR100, BioFroxx, Einhausen, Germany) for 1 h, the slides were incubated with anti‐TFAM (1:50, 22586‐1‐AP, Proteintech), anti‐TOM20 (1:100, 42406S, Cell Signaling Technology), anti‐laminin (1:400, LN, ab11575, Abcam), anti‐CD31 (1:100, ab222783, Abcam), anti‐HGF (1:100, 26881‐1‐AP, Proteintech), anti‐Alix (12422‐1‐AP, Proteintech), anti‐CD63 (1:100, ab217345, Abcam), anti‐α‐SMA (1:100, ab124964, Abcam), anti‐CD81 (1:100, ab109201, Abcam), anti‐EEA1 (1:100, CL488‐68065, Proteintech), anti‐Smad2/3 (1:100, ab202445, Abcam) and anti‐FN (1:100, 15613‐1‐AP, Proteintech) antibodies overnight at 4 °C. After being washed with PBS, the slides were incubated with Alexa Fluor 488‐conjugated goat anti‐rabbit secondary antibody (1:250, ab150077, Abcam), Alexa Fluor 594‐conjugated goat anti‐rabbit secondary antibody (1:250, ab150080, Abcam), or Alexa Fluor 647‐conjugated donkey anti‐mouse secondary antibody (1:250, ab150107, Abcam) as needed at 37 °C for 1 h. Nuclei were stained with DAPI (1 µg mL^−1^, D8417, Sigma‒Aldrich) for 5 min and then washed with PBS. The stained slides were imaged via confocal laser scanning microscopy (Nikon, N‐STORM & A1).

### Isolation of Tissue‐Derived EVs

Tissue EVs were isolated from mouse skeletal muscle, kidney and plasma according to previously reported methods with some modifications.^[^ ^7^ ^]^ Briefly, the mice were sacrificed by an overdose of pentobarbital sodium. For isolation of muscle‐derived EVs from exercise (HIE) or metabolic engineering (OE), the tissues were washed with PBS and then cut into small pieces (≈1 mm^3^) in precooled DMEM. Then, small tissue pieces were digested with collagenase IV (1 mg mL^−1^, 17 104 019, Gibco) and dispase (1 U mL^−1^, D6430, Solarbio, Beijing, China) at 37 °C for 2 h. Afterward, excessive precooled DMEM was used to stop the digestion. For the isolation of plasma‐EVs, mouse plasma was collected and centrifuged at 3000 × rpm for 10 min. After the samples were prepared, they were subjected to a series of centrifugations at 300 × g for 10 min to remove the tissue masses and 2000 × g for 30 min and 10 000 × g for 30 min at 4 °C to remove the cells and other debris. The supernatant was subsequently ultracentrifuged at 120 000 × g for 70 min at 4 °C, after which the EV pellets were washed by resuspending in PBS and repelleting via a second round of ultracentrifugation. Finally, the pure EV pellets were resuspended in sterile PBS and stored at −80 °C for further experiments.

### Characterization of EVs

EVs isolated from tissues or cells were characterized as described previously.^[^ ^50^ ^]^ The morphology of the EVs was examined via transmission electron microscopy (TEM, HT7800, Hitachi, Ltd., Tokyo, Japan). The size distribution of the EVs was analyzed via a nanoparticle tracking analyzer (NTA, Particle Metrix, Meerbusch, Germany). The surface markers of the EVs (Alix, TSG101, CD81 and CD63) were detected via Western blotting. The protein concentration of the EVs was quantified via a micro BCA protein assay kit (CW2011S, CWBIO). The particle/protein ratio was calculated by normalizing the particle number (particles mL^−1^, determined by NTA) to the protein concentration (mg mL^−1^, determined by BCA) of each EV sample (particles per mg of protein), which was calculated as previously reported.^[^ ^4^ ^]^

### Cell Culture and Treatment

Mouse myoblast cell lines (C2C12) were cultured in Dulbecco's modified Eagle's medium (C11995500BT, DMEM, Gibco, CA, USA) supplemented with 10% fetal bovine serum (FBS, FSP500, Excell Biological Technology Co., Ltd. Shanghai, China) and 1% penicillin/streptomycin (C0222, Beyotime Biotechnology). C2C12 cells were exposed to 0.4 mM H_2_O_2_ (0.4 mM; Sigma‒Aldrich, USA) for 48 h to induce oxidative stress injury and then treated with muscle‐derived EVs (≈6 × 10^9^ particles mL^−1^). To impede mitochondrial function, 10 µM FCCP (HY‐100410, MedChemExpress, Monmouth Junction, NJ, USA) was used, and 2 µM C75 (C5490, Sigma‒Aldrich, USA) was used to inhibit the lipid synthesis pathway. Mouse primary renal proximal tubular cells (PTECs) were isolated from mouse kidney tissues as previously reported,^[^ ^51^ ^]^ and PTECs were cultured in DMEM/F12 (C11339500BT, Gibco) supplemented with 1% penicillin−streptomycin and 10% FBS. PTECs were exposed to 10 ng mL^−1^ TGFβ1 (Recombined Mouse, Sino Biological, Inc., Beijing, China) for 48 h to induce a fibrotic phenotype and were then cotreated with muscle EVs (≈6 × 10^9^ particles mL^−1^) or 50 ng mL^−1^ rHGF (positive control, Recombined Mouse, Sino Biological, Inc.). All the cells were incubated in a humidified atmosphere at 37 °C with 5% CO_2_.

### Quantitative Real‐Time PCR

Total RNA was extracted from cells or tissues via TRIzol reagent (15596018CN, Thermo Fisher Scientific) and reverse‐transcribed into cDNA via a cDNA synthesis kit (R323‐01, Vazyme, Nanjing, China). Real‐time polymerase chain reaction (real‐time PCR) was performed on a CFX96 real‐time PCR detection system (Bio‐Rad, Hercules, CA, USA) with SYBR Green (Q712, Vazyme). The primers used in this study are listed in Table S1, Supporting Information. The data were analyzed via Bio‐Rad CFX Manager software, and the relative change in mRNA expression was calculated via the delta‒delta Ct method with rps18 as an internal reference gene.

### Mitochondrial Membrane Potential Assay

After treatments, the C2C12 cells were stained with 100 nM Image‐iT TMRM (I34361, Invitrogen, USA) and Hoechst 33 258 (C1011, Beyotime Biotechnology, Shanghai, China) at 37 °C for 30 min. After washing with PBS, representative images were observed by via confocal microscopy (Nikon, N‐STORM & A1, Tokyo, Japan).

### Isolation of Cell‐Derived EVs

Cell‐derived EVs were isolated from conditioned medium via a differential centrifugation method as previously reported.^[^ ^50^ ^]^ EV‐depleted FBS was prepared by ultracentrifugation (120 000 × g, 18 h, 4 °C) as described previously.^[^ ^50^ ^]^ C2C12 cells were cultured in DMEM containing 10% EV‐depleted FBS for 24 h, and their culture media were collected for EV isolation. In brief, the collected medium was centrifuged at 300 × g for 10 min, 2000 × g for 30 min and 10 000 × g for 30 min at 4 °C to remove cells and other debris, followed by ultracentrifugation at 120 000 × g for 70 min at 4 °C in an SW32Ti rotor (Optima XPN‐100, Beckman Coulter, Brea, CA, USA) to obtain EVs. After washing with PBS, the EV pellets were centrifuged at 120 000 × g for 70 min at 4 °C again. The purified EVs were resuspended in PBS and stored at −80 °C until further use.

### Mouse CKD Model and Treatments

Male mice (8–10 weeks, Permit No. 20211551A) were randomly divided into four groups (n = 6): the Ctrl (WT) group, Ctrl (OE) group, CKD (WT) group, and CKD (OE) group. The bilateral ureteral obstruction (UUO)‐induced CKD mouse model was established by ligating the left ureter in the mice as previously described.^[^ ^52^ ^]^ The body weights and grip strengths of the mice were measured before surgery. The mice were anesthetized via i.p. injection of sodium pentobarbital (50 mg kg^−1^), followed by ligation of the left ureter with 7–0 silk. The Ctrl mice underwent the same procedure without ureter ligation.

Mouse muscle TFAM overexpression (OE) was induced by intramuscular (im) injection of rAAV2/9‐CMV‐*tfam* vectors (≈1.8 × 10^12^ genomic particles, Hanbio Biotechnology, Inc., Shanghai, China) in the tibialis anterior (TA) and gastrocnemius muscles at four independent points (15 µL/point), whereas mice in the wild‐type (WT) group were injected with equal amounts of rAAV2/9‐CMV‐null vectors. The mice in the OE group were injected with AAV on day 7 before UUO surgery. On day 30 after UUO surgery, the body weight and grip strength of the mice in each group were measured. After that, the mice in each group were sacrificed by an overdose of anesthesia, and their muscle and kidney tissues were collected for further tests.

### RNA Sequencing Assay of Muscles

After the indicated treatments, mouse muscle tissues were collected for RNA sequencing (RNA‐seq) analysis. In brief, total RNA was extracted from tissue samples via TRIzol, and genomic DNA was removed via DNase I (Takara, Shiga, Japan). The RNA‐seq transcriptome library was constructed via a TruSeq RNA Sample Kit (Illumina, San Diego, CA, USA), and the paired‐end RNA‐seq sequencing library was sequenced on an Illumina HiSeq Xten/NovaSeq 6000 sequencer by Shanghai Majorbio Biopharm Technology Co., Ltd. (Shanghai, China). The expression levels of the transcripts were calculated via the transcripts per million reads (TPM) method. DEGs with a fold change (FC) > 1.2 and *p* value < 0.05 were identified via DESeq2 on the basis of read counts. Subsequently, clusterProfiler (version 4.8.1) was used to analyze the functional enrichment of the DEGs, including GO and KEGG pathway enrichment. Finally, ggplot2 (version 3.4.4) was used to visualize the analysis results, such as by generating heatmaps and volcano plots in the R script (version 4.2.3).

### Proteomic Analysis of Tissue EVs

Label‐free proteomic analysis of tissue EV samples was performed via a liquid chromatography‒tandem mass spectrometry (LC‒MS/MS) detection system (Thermo Fisher Scientific) as previously described.^[^ ^7^ ^]^ In brief, total proteins were extracted from EVs and refined into peptides. Then, 500 ng of each peptide sample was dissolved in 0.1% FA and separated on a ReproSil‐Pur C18‐AQ column (Dr. Maisch HPLC GmbH, Ammerbuch, Germany) over a 78 min gradient at a flow rate of 300 nL min^−1^. The raw data files from the Orbitrap Fusion Lumos MS (Thermo Fisher Scientific) were searched against the Rat Swiss‐Prot database via MaxQuant (v. 1.5.3.8; Max Planck Gesellschaft, Munich, Germany). Label‐free proteome quantitation (LFQ) intensities were used for quantitation of the EV proteins. DEPs with a fold change (FC > 1.2 and p‐adjust < 0.05) between two groups were assessed via a free online platform (https://www.omicsolution.com/wkomics/wkold/). DEPs among the three groups were assessed via limma analysis with one‐way ANOVA tests (p‐adjust < 0.05), which uses linear models to assess differential expression in multifactor (group) experiments. Gene Ontology (GO) analysis was performed via STRING (https://cn.string‐db.org/), and a false discovery rate (FDR) < 0.05 was considered significant. To identify the source of EVs, we took advantage of public scRNA‐seq data and our LC–MS/MS results as previously reported. To quantify the contributions of different cell types to the EV proteome, we applied the CIBERSORT algorithm. Using the signature matrix derived from scMappR, we performed deconvolution on the proteomic data of EVs as previously reported.^[^ ^42^ ^]^

### In Vivo EV Biodistribution Assay

The isolated EVs were labeled with Cy7 dye (sulfo‐Cy7‐NHS ester, X‐CL‐1397, New Research Biosciences Co., Ltd., Xi'an, China) or DID (D307, Thermo Fisher Scientific) as previously reported,^[^ ^50^ ^]^ and the unbound dyes were removed via ultracentrifugation. One hundred microliters of dye‐labeled EVs (≈3.9 × 10^10^ particles mouse^−1^) or an equal amount of free dye (negative control) was administered to the mice via intramuscular injection at four different sites. At 4 or 24 h after EV injection, the mice were sacrificed by an overdose of anesthesia, and the hearts, lungs, livers, kidneys, spleens and lower limbs were collected and observed via an optical imaging system (IVIS Spectrum, PerkinElmer, Waltham, MA, USA). The fluorescence intensity was quantified via Analyze software (version 12.0, PerkinElmer). In addition, frozen kidney or muscle tissue sections from the mice were labeled with LTL (1:500, FL‐321, Vector Laboratories Inc., CA, USA), anti‐laminin (LN, 1:400, ab11575, Abcam) and DAPI (1 µg mL^−1^, Sigma, USA), and the stained sections were subsequently observed under a confocal microscope (Nikon, N‐STORM & A1).

To label endogenous muscle tissue EVs, mouse muscle was injected with rAAV2/9‐ZsGreen vectors (1.9 × 10^12^ genomic particles; Hanbio Biotechnology, Inc.) or AAV‐null vectors. On day 7 after injection, the CKD group underwent UUO surgery, and the Ctrl mice underwent the same procedure without ureter ligation. On day 14 after surgery, EV samples from muscle, kidney or plasma were isolated via centrifugation and ultracentrifugation. EVs with GFP‐positive signals were detected via nanoFCM (Fuliu Biology Science and Technology Co., Ltd., Xiamen, China).

### TEM Assay of Muscle Mitochondria

For the preparation of tissue samples for TEM, fresh mouse muscle tissues were fixed with 2.5% glutaraldehyde solution (R20515, Yuanye Bio‐Technology Co., Ltd., Shanghai, China), dehydrated, and embedded in resin. The embedded tissues were cut into ultrathin sections and stained with 2% uranyl acetate and Reynolds lead citrate. The morphology of the mitochondria in the muscle tissues was observed via TEM (H‐600, Hitachi, Ltd., Tokyo, Japan) at a voltage of 75 kV.

### Histological Examination

Renal or muscle tissues were perfusion fixed in 4% paraformaldehyde, embedded in paraffin, cut into 5 µm thick sections and further processed for H&E, Masson and immunohistochemistry (IHC) staining. For IHC staining, tissue sections were subjected to a series of deparaffinization, rehydration, and antigen retrieval processes. After inactivation of endogenous peroxidase with 3% hydrogen peroxide, the sections were blocked with 1% BSA, incubated with diluted anti‐ICAM‐1 (1:200, 10831‐1‐AP, Proteintech, Wuhan, China), anti‐F4/80 (1:400, 28463‐1‐AP, Proteintech), anti‐MuRF1 (1:200, 55456‐1‐AP, Proteintech, Wuhan, China) and anti‐α‐SMA (1:200, ab7817, Abcam, Cambridge, MA, USA) antibodies overnight at 4 °C and then stained with HRP‐conjugated secondary antibodies (1:2000, ab205718, Abcam) and 3,3′‐diaminobenzidine substrates. Images of the stained sections were scanned via a digital pathology section scanner (NanoZoomer S360, Hamamatsu Photonics, Japan) and quantified via ImageJ software (NIH).

### Statistical Analysis

The quantitative data are expressed as the means ± standard deviations (SDs). Statistical analysis was performed via GraphPad Prism software (version 9.0.0; IBM Corporation, USA). The differences between groups were determined by t tests (for two‐group comparisons) or one‐way analysis of variance (ANOVA) (for more than two group comparisons), and *p* < 0.05 was considered statistically significant.

## Conflict of Interest

The authors declare no conflict of interest.

## Supporting information



Supporting Information

## Data Availability

The data that support the findings of this study are available from the corresponding author upon reasonable request.
