# Supplementary material for: In Vivo Reprogramming of Tissue‐Derived Extracellular Vesicles for Treating Chronic Tissue Injury Through Metabolic Engineering
Source: Adv Sci (Weinh). 2025 Mar 31;12(21):2415556. doi: 10.1002/advs.202415556 (PMC12140305; doi:10.1002/advs.202415556)
Supplement: Supplementary file 1 — Supporting Information [file ADVS-12-2415556-s001.docx]

**Supplementary materials**

**In Vivo Reprogramming of Tissue-derived Extracellular Vesicles for Treating Chronic Tissue Injury through Metabolic Engineering**

Meng Zhao**^#^**^1,2^, Shuyun Liu**^#^**^1^, Yizhuo Wang^1^, Peng Lou^1^, Ke Lv^1^, Tian Wu^1^, Lan Li^1^, Qianyi Wu^2^, Jiaying Zhu^2^, Yanrong Lu^1^, Meihua Wan^3^, Jingping Liu^1^*

^1^ Department of General Surgery and NHC Key Laboratory of Transplant Engineering and Immunology, Frontiers Science Center for Disease-related Molecular Network, West China Hospital, Sichuan University, Chengdu 610041, China

^2^ Department of Emergency, Guizhou Provincial People's Hospital, Guiyang 550002, China

^3^ West China Center of Excellence for Pancreatitis, Institute of Integrated Traditional Chinese and Western Medicine, West China Hospital, Sichuan University, Chengdu 610041, China

**^#^** Co-first authors who contributed equally to this work.

* Corresponding Author: Jingping Liu

Email: liujingping@scu.edu.cn

Address: NHC Key Laboratory of Transplant Engineering and Immunology, West China Hospital, Sichuan University, No. 2222 Xinchuan Road, Chengdu 610041, China.

Tel: +86-28-85164029, Fax: +86-28-85164030

***In Vivo* Biodistribution of DID-labeled EVs in Mice**

Lipophilic near-infrared dyes (DID, Invitrogen) were used to label the EVs from myoblasts, and the unbound dyes were removed via ultracentrifugation. One hundred microliters of DID-labeled EVs (DID-EVs, ~3.9 × 10^10^ particles/mouse) or an equal amount of free DID (negative control) or PBS was administered to CKD model or Ctrl mice via intramuscular injection at four different sites. At 4 h or 24 h after the injection, the mice were sacrificed by an overdose of anesthesia, and the hearts, lungs, livers, kidneys, spleens and lower limbs were collected and observed on an optical imaging system (IVIS Spectrum, PerkinElmer, Waltham, MA, USA).

**Cell migration assay**

The cell migration ability was assessed via a cell scratch assay. In brief, myoblasts were seeded in 24-well plates, and a straight line was scratched with a sterile 1 mL pipette tip when the cells reached 90% confluence. Then, the cells were treated with EVs (~6 × 10^9^ particles/mL) and cultured in FBS-free DMEM for 48 h. Images of the narrow wound-like gaps were captured with an inverted microscope (Eclipse TS100, Nikon, Japan), and the wound closure ratio was analyzed with ImageJ software (NIH, Bethesda, MD, USA).

**Cellular uptake assay of EVs**

The isolated EVs were labeled with PKH26 (Sigma‒Aldrich, USA) fluorescent dye as previously reported^1^. PKH26-labeled EVs (~6 × 10^9^ particles/mL) were added to myoblasts or PTECs cells in DMEM at 37°C for 6 h. After washing with PBS, the cells were fixed with 4% paraformaldehyde for 15 min and then stained with FITC-phalloidin (Yesen, Shanghai, China) for 15 min at room temperature. Nuclei were visualized by staining with DAPI (Sigma, USA). Images of the stained cells were obtained via a confocal laser scanning microscope (Nikon, N-STORM & A1).

***In Vivo* biosafety and immunogenicity of the AAV-TFAM vector in Mice**

Male mice were randomly divided into two groups: the Ctrl (WT) group (n = 6), and the OE group (n = 6). Mouse muscle TFAM overexpression (OE) was induced by intramuscular (im) injection of rAAV2/9-CMV-*tfam* vectors (1.8 × 10^12^ genomic particles, Hanbio Biotechnology, Inc., Shanghai, China) in the tibialis anterior (TA) and gastrocnemius muscles at four independent points (15 μL/point), whereas mice in the wild-type (WT) group were injected with equal amounts of PBS. On day 30 after AAV administration, the mice in each group were sacrificed by an overdose of anesthesia, and their blood and major organs/tissues (heart, liver, spleen, lung, kidney and muscle) were collected for further tests.

**Clinical biochemical tests**

Renal function indicators (CREA and UREA) and liver function indicators (ALT and AST) levels in the serum samples of the mice were analyzed on a biochemistry analyzer (ci16200, ARCHITECT Diagnostics, Abbott, Lake Forest, IL, USA) using appropriate commercial kits.

**Analysis of serum IL-6**

Blood samples were centrifuged at 3000 rpm for 10 min, and IL-6 concentrations in the serum were measured via a mouse IL-6 One Step ELISA Kit (S0C3019, Starter Biotechnology Co., Ltd, Hangzhou, China) according to the manufacturer’s instructions. Briefly, the diluted serum samples were incubated with a premixed cocktail of antibody-conjugated beads in a 96-well plate for 45 min, The sample-loaded plate was washed with buffer and incubated with TAB substrate and stop buffer, and the protein concentration was measured on a Luminex 200 analyzer (Luminex, Austin, TX, USA) according to the manufacturer’s instructions.

**Tim4-affinity purification of muscle-derived EVs**

Muscle EVs were isolated via the MagCapture Exosome Isolation Kit PS Ver.2 (290-84103, FUJIFILM Wako, Japan) following the manufacture’ instructions. Briefly, the mice were sacrificed by an overdose of pentobarbital sodium. The muscle tissues were washed with PBS and then cut into small pieces (~1 mm^3^) in precooled DMEM and then, small tissue pieces were digested with collagenase IV (1 mg/mL, 17104019, Gibco) and dispase (1 U/mL, D6430, Solarbio, Beijing, China) at 37°C for 2 h. Afterward, excessive precooled DMEM was used to stop the digestion. These samples were subjected to a series of centrifugations at 300 × g for 10 min to remove the tissue masses, 2000 × g for 30 min and 10000 × g for 30 min at 4°C to remove the cells and other debris. After preparation of the Biotin Capture Magnetic Beads and Biotin-labeled Exosome Capture mixture, the prepared samples were added to beads tubes and then rotated for 1 h at room temperature. Afterward, the bound EVs were eluted with Exosome Elution Buffer.

**Supplemental Results**

**
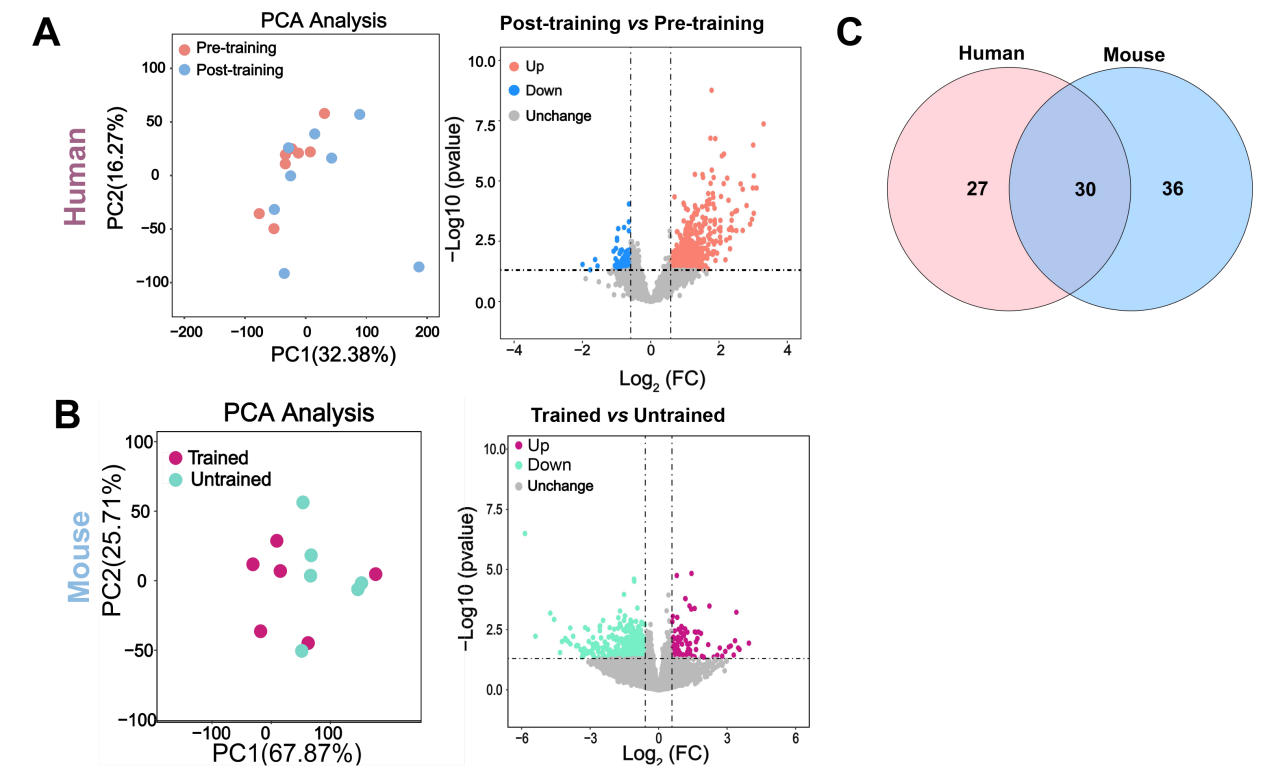
**

**Figure S1. Effect of HIE training on skeletal muscle gene expression.** (A-B) PCA scatterplot of different groups based on GEO data and volcano plots showing the discrepancies between different groups in human samples (n = 8 subjects) and mouse samples (n = 6 mice). (C) Venn diagram representing the number of unique and overlapping genes.

**
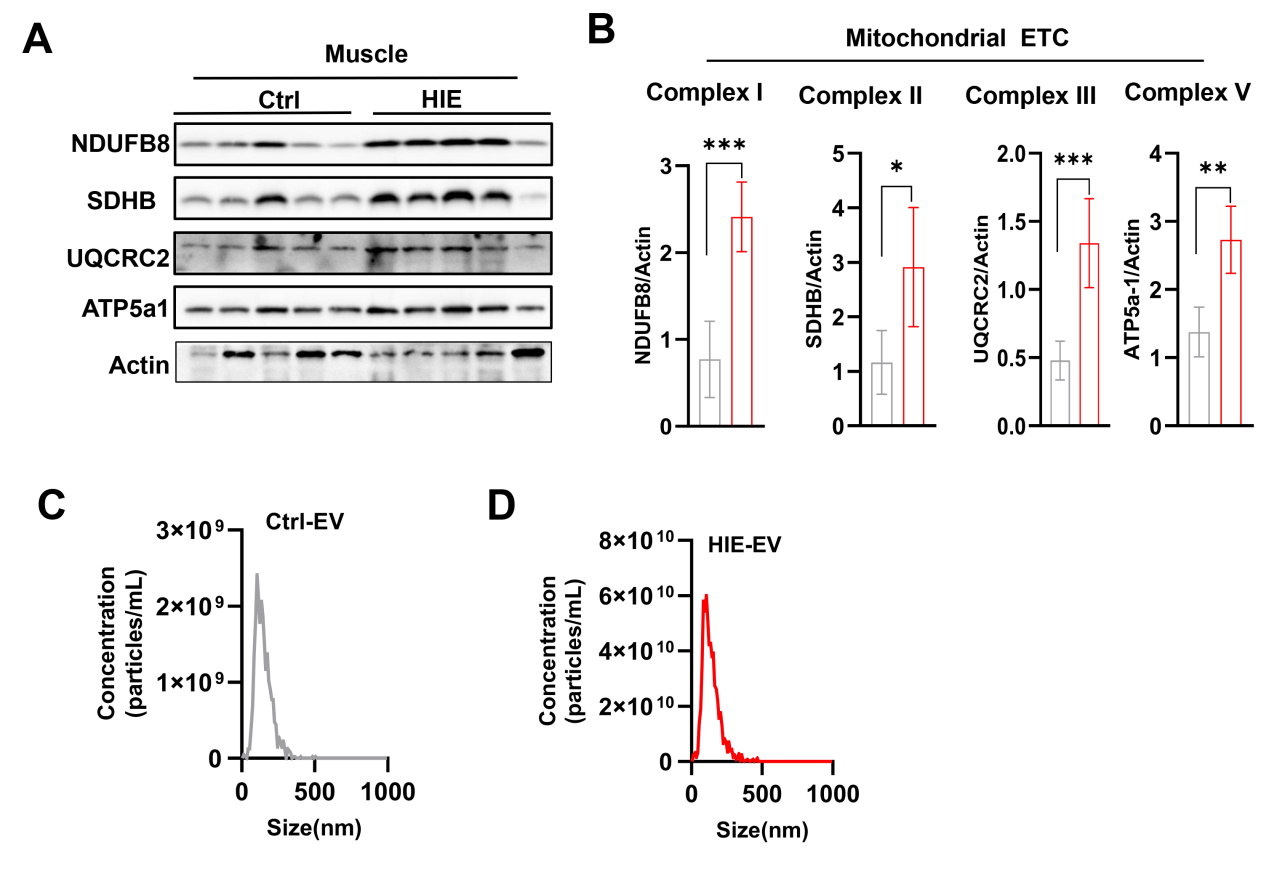
**

**Figure S2. HIE training enhanced mitochondrial biogenesis in the muscle of healthy mice.** (A-B) Western blot and quantification of the mitochondrial ETC proteins (NDUFSB8, SDHB, UQCRC2 and ATP5a-1) in the muscle of the mice (n = 5 mice; * p < 0.05, ** p < 0.01, *** p < 0.001). (C-D) Size distribution and particle distribution of muscle-derived EVs determined by NTA.


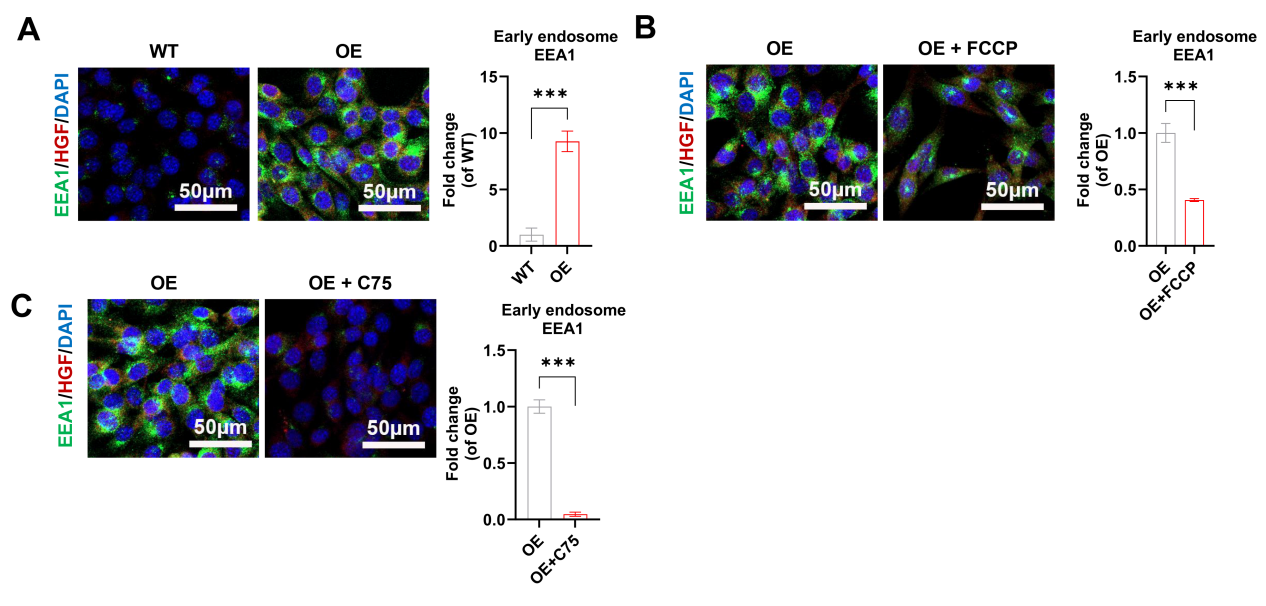


**Figure S3** (A-C) Representative micrographs and quantification of EEA1 and HGF IF staining in plasmid transfected-myoblasts after treatment with or without FCCP/C75 for 24 h (scale bar = 50 μm, n = 3 biological replicates, *** P < 0.001).


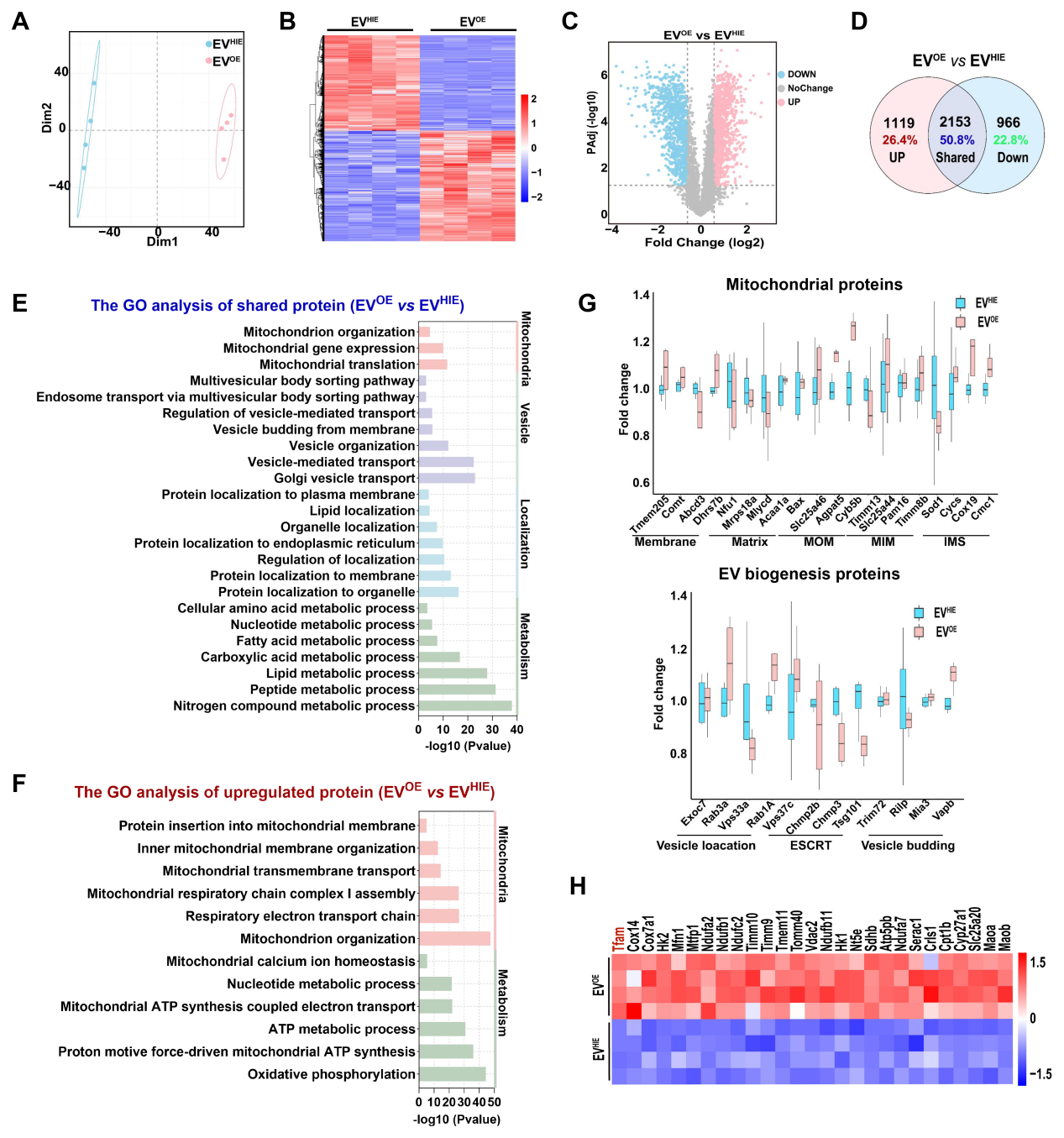


**Figure S4. Proteomic analysis of muscle tissue-derived EVs from healthy mice subjected to exercise (EV^HIE^) or metabolic engineering (EV^OE^).** (A-C) PCA scatter plot (A), heatmap (B) and volcano plots (C) of different groups based on proteomic data showing the DEPs in muscle EVs (n = 4 mice, FC > 1.5 and p-adjust < 0.05). (D) Venn diagram showing the upregulated, shared, and downregulated protein numbers between the EV^OE^ group and the EV^HIE^ group. (E) GO enrichment analyses showing the shared proteins between the EV^OE^ group and the EV^HIE^ group. (F) GO enrichment analyses showing the upregulated proteins between the EV^OE^ group and the EV^HIE^ group. (G) The bar graph showed the shared proteins which related to mitochondrial component (upper panel) and EV biogenesis (lower panel) between the EV^OE^ group and the EV^HIE^ group. (H) Heatmap showing the upregulated proteins which related to mitochondrial oxidative phosphorylation and metabolism between the EV^OE^ group and the EV^HIE^ group.


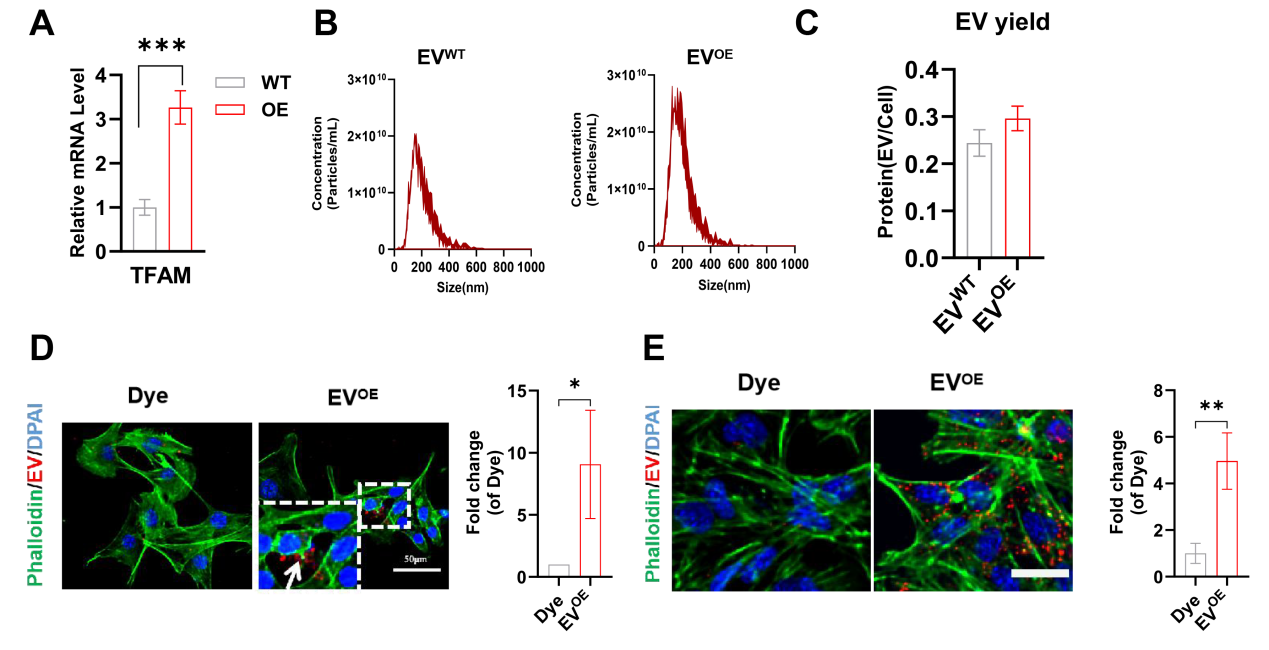


**Figure S5. Generation and characterization of myoblast-derived EVs in vitro.** (A) The relative mRNA and protein expression levels of TFAM in myoblasts transfected with plasmid (n = 3 biological replicates; *** p < 0.001). (B) Size distribution was detected via NTA. (C) Comparison of EV yield (quantified by cell protein and EV protein) in myoblasts transfected with plasmids for 24 h (n = 3 biological replicates). (D-E) Representative images and quantification of PKH26-labeled EV (red) uptake in myoblasts or PTECs stained with FITC-phalloidin (green) and DAPI (blue) (scale bar = 50 μm; n = 3 biological replicates; *** p < 0.001).

**
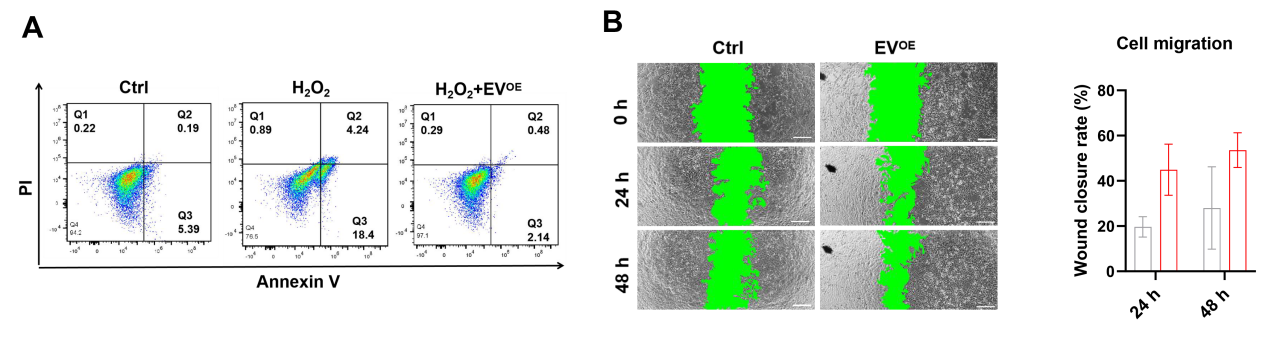
**

**Figure S6. Evaluation of the biological effects of muscle-derived EVs *in vitro*.** (A) Detection of apoptotic cells via flow cytometry with Annexin V/PI staining. Damaged myoblasts (induced by 0.4 mM H_2_O_2_) were treated with EV^OE^ (~6 × 10^9^ particles/mL) for 48 h. (B) Representative cell migration images and quantification of the cell migration ratio in myoblasts treated with EV^OE^ (scale bar = 500 µm; n = 3 biological replicates).


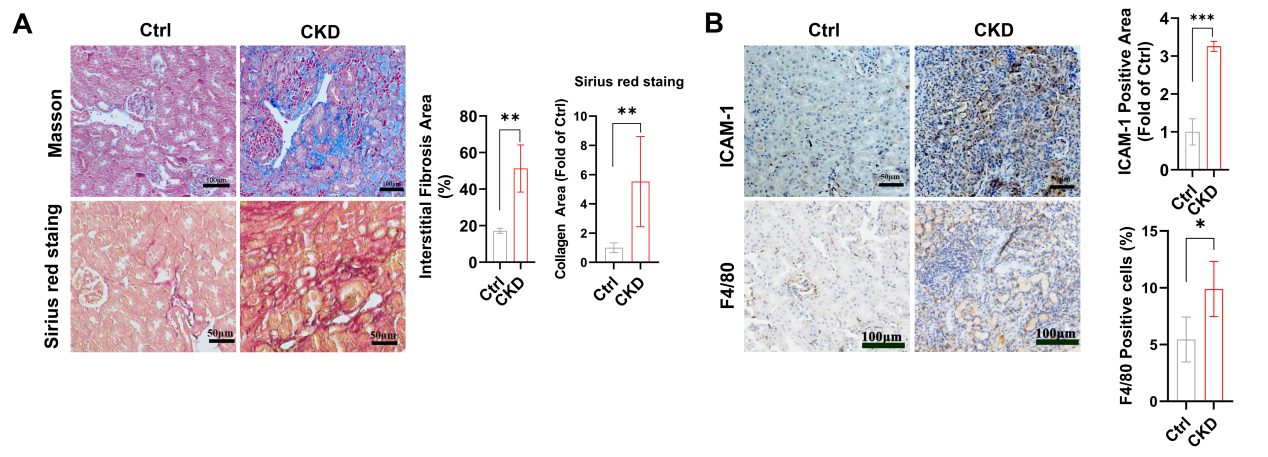


**Figure S7. Evaluation of renal fibrosis and inflammation in CKD mice.** (A) Representative images and quantification of Masson and Sirius red staining of kidney tissues on day 30 after surgery (scale bar = 100 μm, n = 6 mice, ** p < 0.01). (B) Representative images and quantification of IHC staining (ICAM-1 and F4/80) of kidney tissues on day 30 after surgery (scale bar = 50 μm for ICAM-1, scale bar = 100 μm for F4/80, n = 6 mice, * p < 0.05, *** p < 0.001).


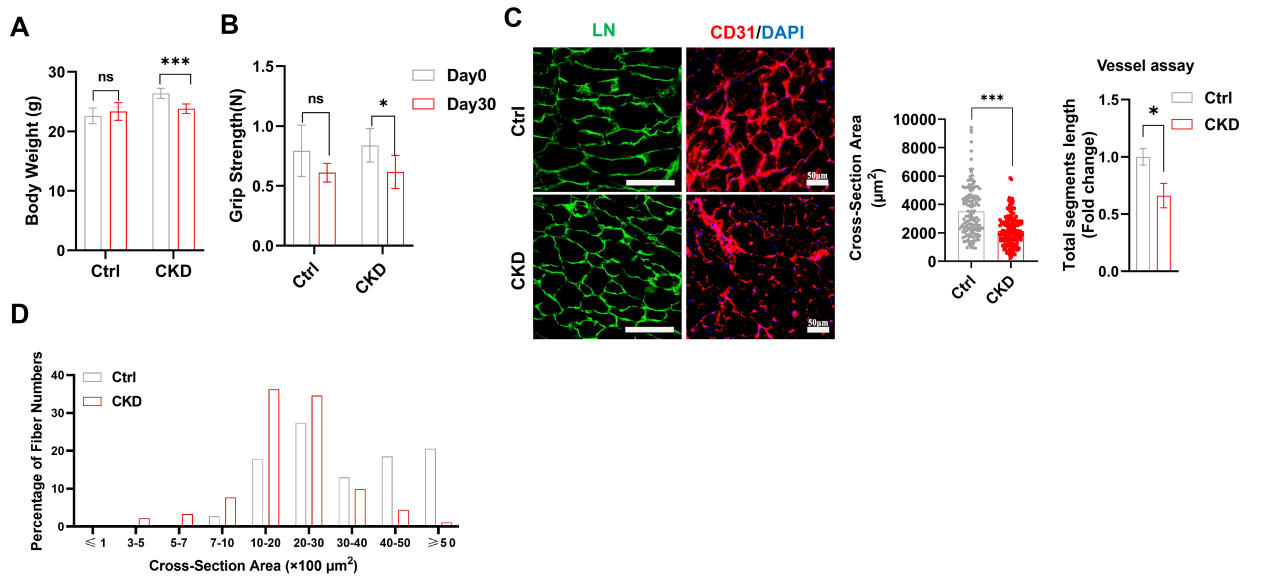


**Figure S8. Evaluation of muscle and vascular injury in CKD mice.** (A-C) The graphs show body weight (A), grip strength (B), cross-sectional area and (C) CD31 (scale bar = 50 μm) and LN (scale bar = 100 μm) IF staining analysis (n = 6 mice; * p < 0.05,*** p < 0.001). (D) The bar graph shows the frequency distribution of fiber cross-sectional areas in CKD mice on day 30 after surgery.


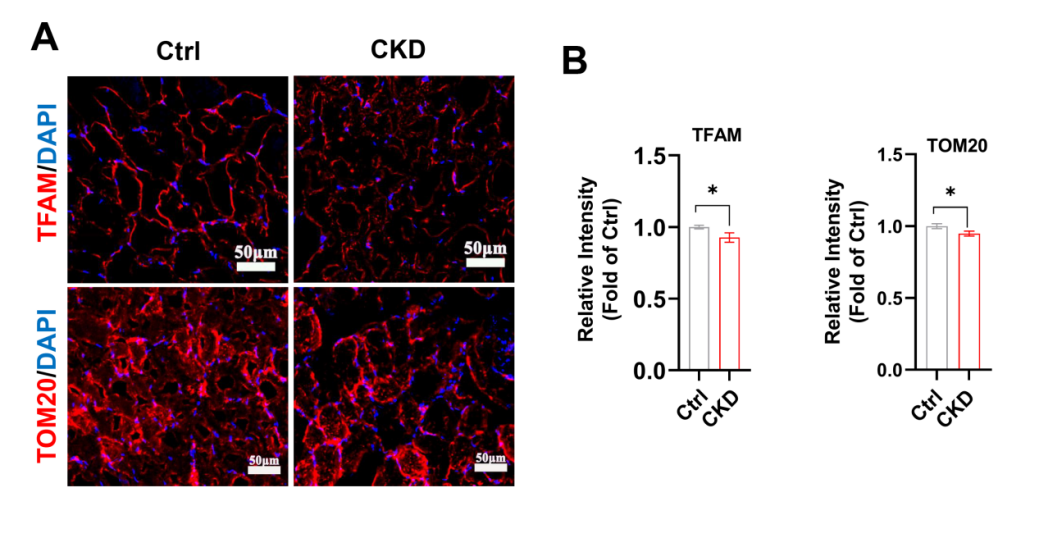


**Figure S9. Evaluation of muscle mitochondrial injury in CKD mice.** (A-B) Representative images and quantification of IF staining (TFAM and TOM20) in muscle on day 30 after surgery (n = 6 mice, * p < 0.05).


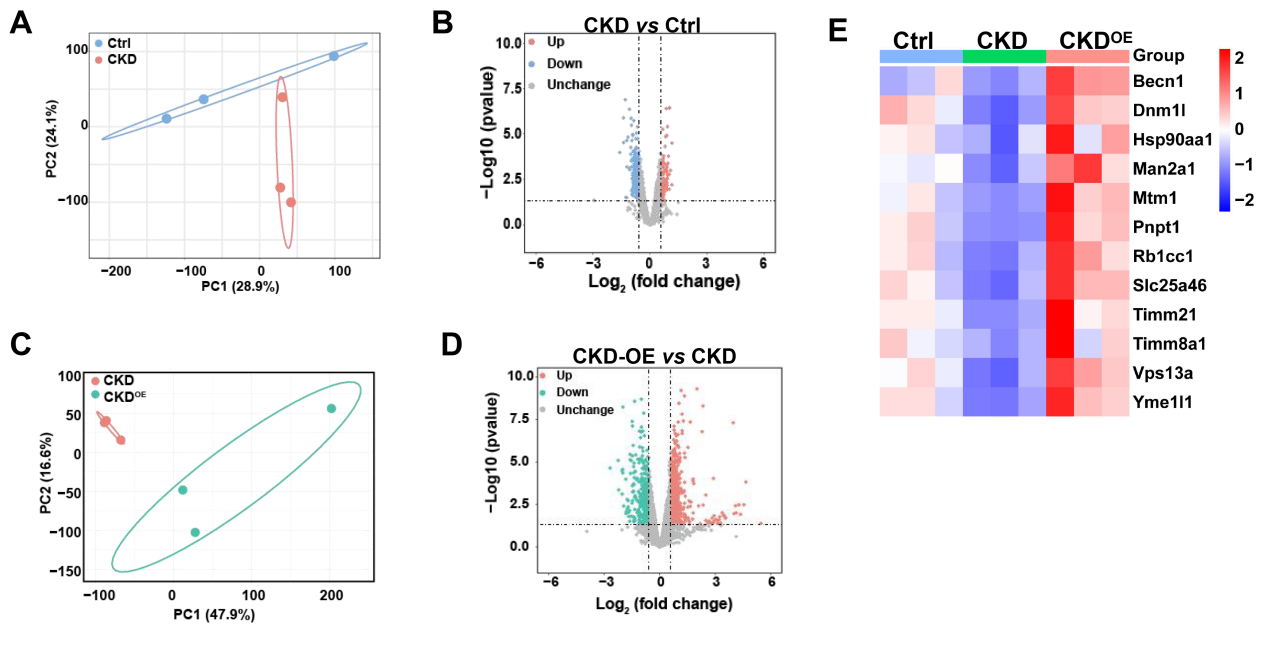


**Figure S10. RNA-seq analysis of muscle from CKD mice after the administration of rAAV-CMV-*tfam*.** (A-D) PCA scatter plot and volcano plots of different groups showing the DEGs between the two groups (n = 3 mice, FC > 1.2, p < 0.05). (E) Heatmap displaying variations in mitochondria, muscle tissue development, vesicle organization and autophagy between the two groups (n = 3 mice).


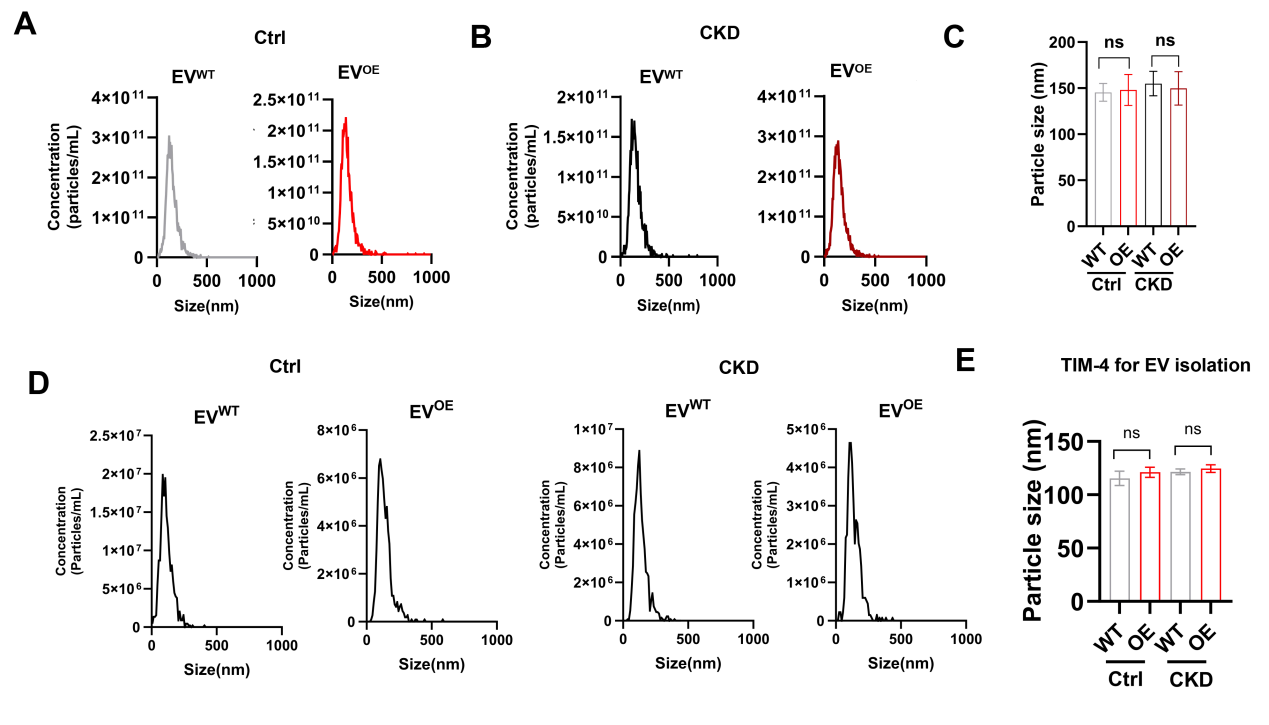


**Figure S11. Detection of the size distribution of muscle-derived EVs.** (A-B) EVs were isolated from the muscle of mice injected with AAV, after which the particle concentration and size distribution were detected via NTA. (C) Size distributions of EVs measured via NTA (n = 6 mice). (D) EVs were isolated from the muscle of mice injected with AAV via MagCapture Exosome Isolation Kit, after which the particle concentration and size distribution were detected via NTA. (E) Size distributions of EVs measured via NTA (n = 6 mice).


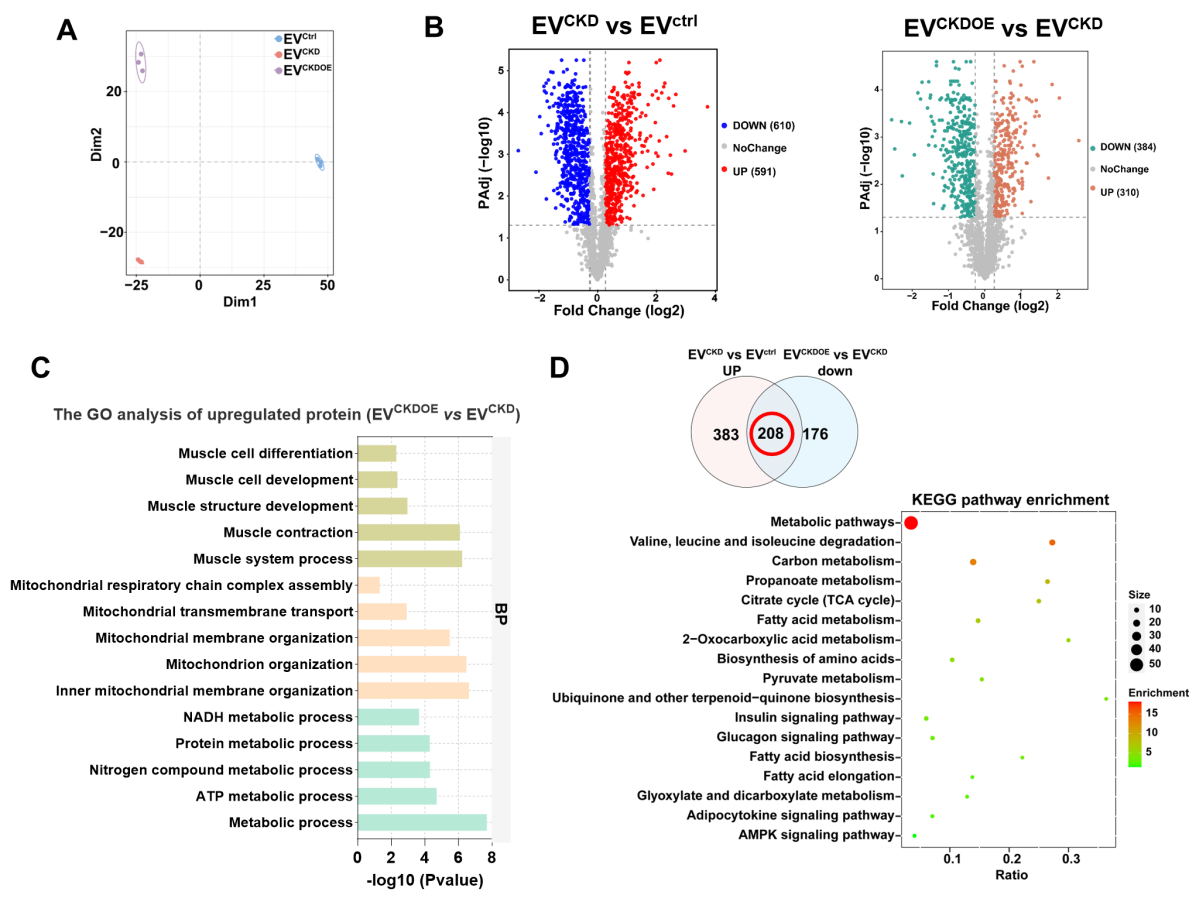


**Figure S12. Proteomic analysis of muscle EVs isolated from CKD mice.** (A-B) PCA scatter plot and volcano plots of different groups based on proteomic data showing the DEPs in muscle EVs (n = 3 mice, FC > 1.2 and p-adjusted < 0.05). (C) GO enrichment analysis showing the upregulated proteins in the EV^CKDOE^ group vs EV^CKD^ group. (D) KEGG enrichment analysis showing the DEPs that were upregulated in the EV^CKD^ group vs EV^Ctrl^ group and downregulated in EV^CKDOE^ group vs the EV^CKD^ group.


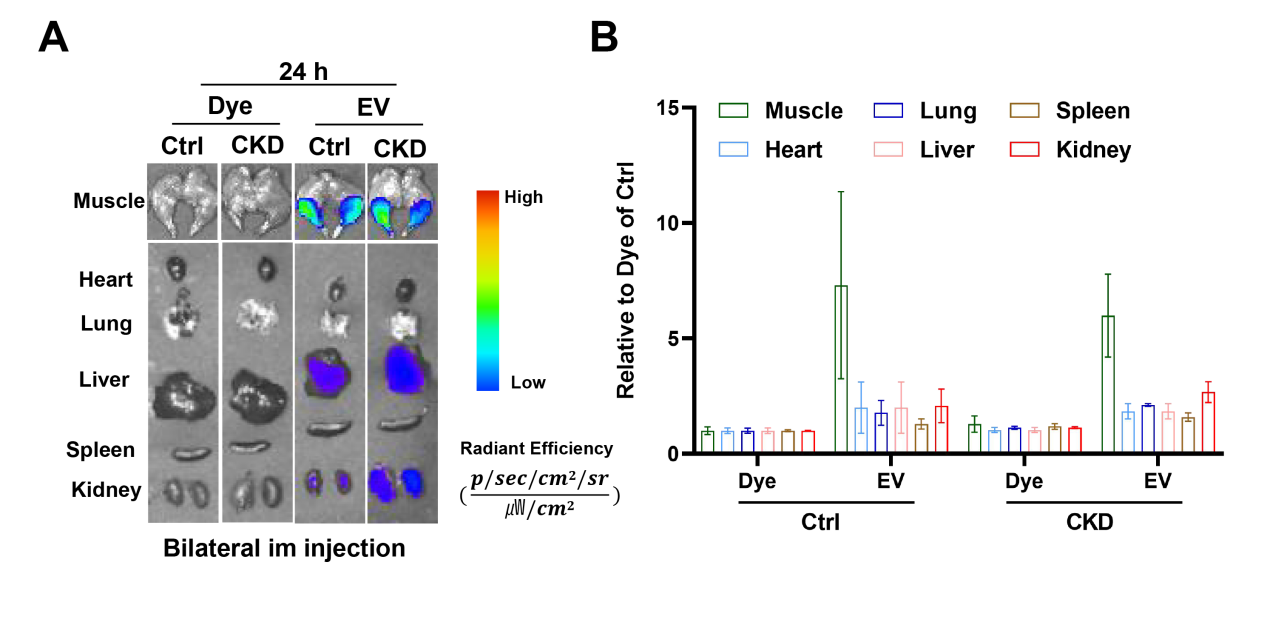


**Figure S13. *In vivo* distribution of EVs in mice.** (A-B) Representative IVIS images and quantification analysis of Cy7-labeled EV biodistribution in different organs (muscle, heart, lung, liver, spleen and kidney) of CKD mice at 24 h via bilateral intramuscular injection (n = 3 mice).


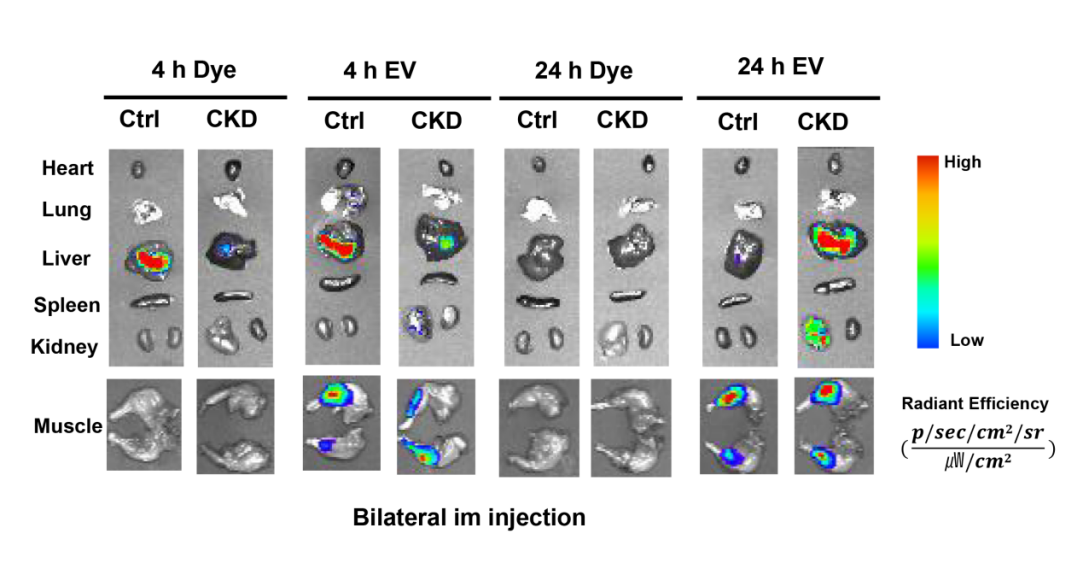


**Figure S14.** Representative IVIS images of DID-labeled EV biodistribution in different organs (muscle, heart, lung, liver, spleen and kidney) of mice at 4 h and 24 h via bilateral intramuscular injection.


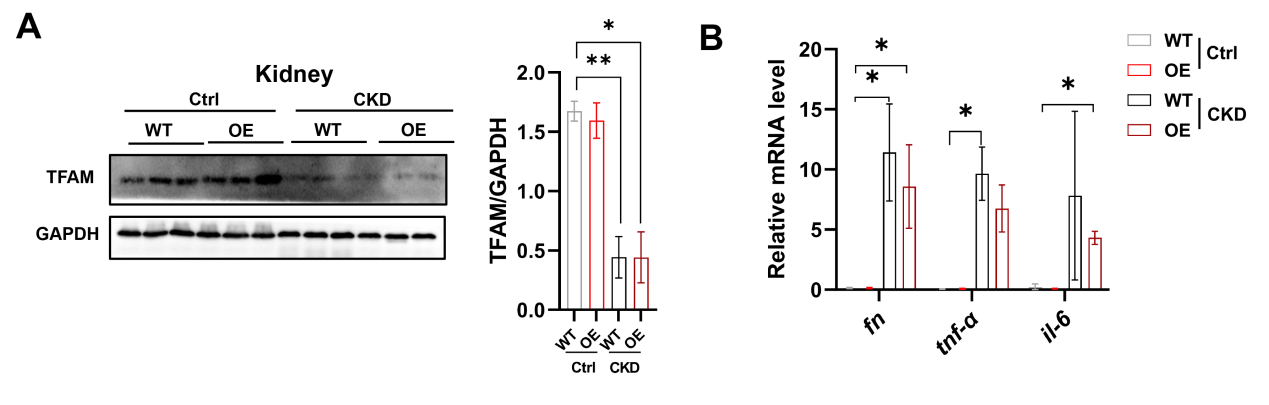


**Figure S15. Detection of TFAM and cytokine expression in mouse kidneys.** (A) Western blot and quantification analysis of TFAM in the kidneys of the mice (n = 3 mice; * p < 0.05, ** p < 0.01). (B) qPCR analysis of gene (tnf-α, il-6, and fn) expression in the kidneys of Ctrl or CKD mice after the administration of AAV (n = 6 mice; * P < 0.05, ** P <0.01).

**
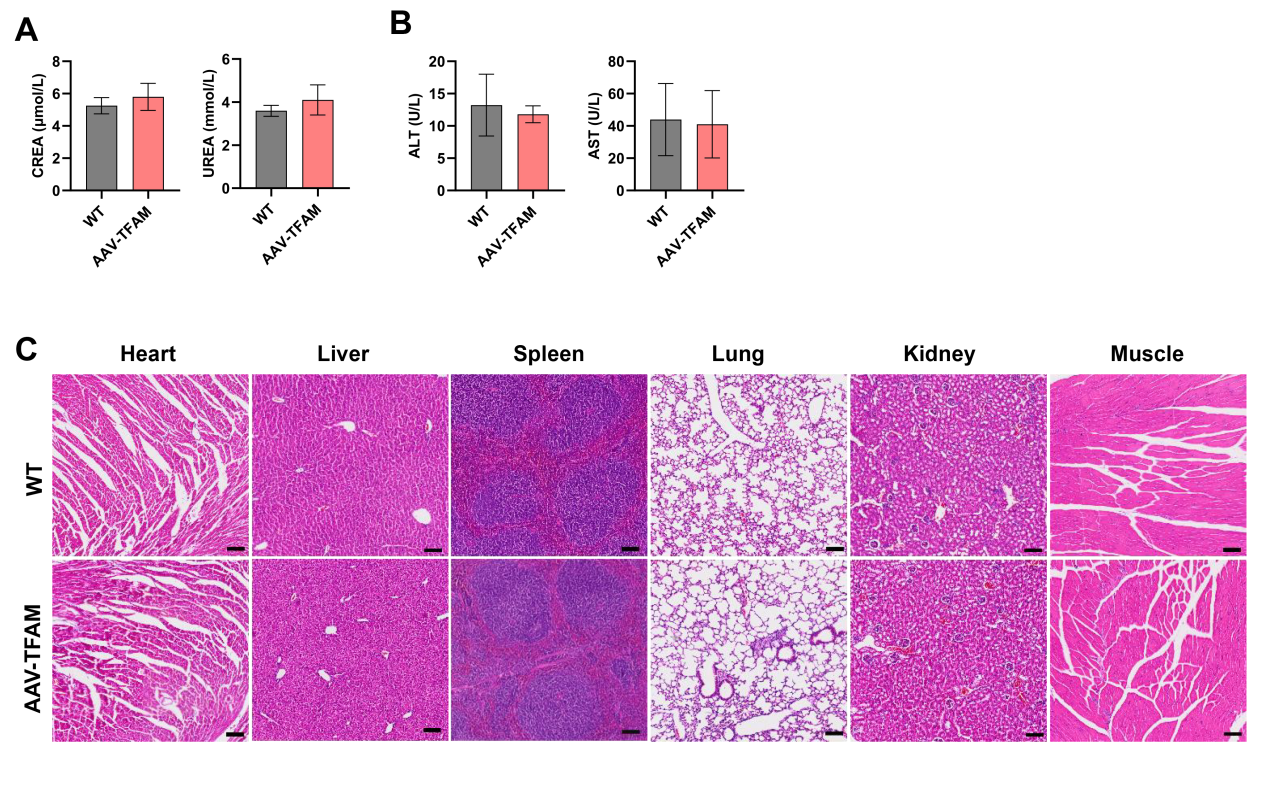
**

**Figure S16 *In vivo* biosafety evaluation of AAV-TFAM vector.** (A-B) Renal function indicator (CREA and UREA) and liver function indicator (ALT and AST) levels of mice at 30 days after AAV-TFAM vector administration (n = 6 mice). (C) Representative H&E staining images of heart, liver, spleen, lung, kidney and muscle tissues from the mice in each group (scale bar = 100 µm).

**
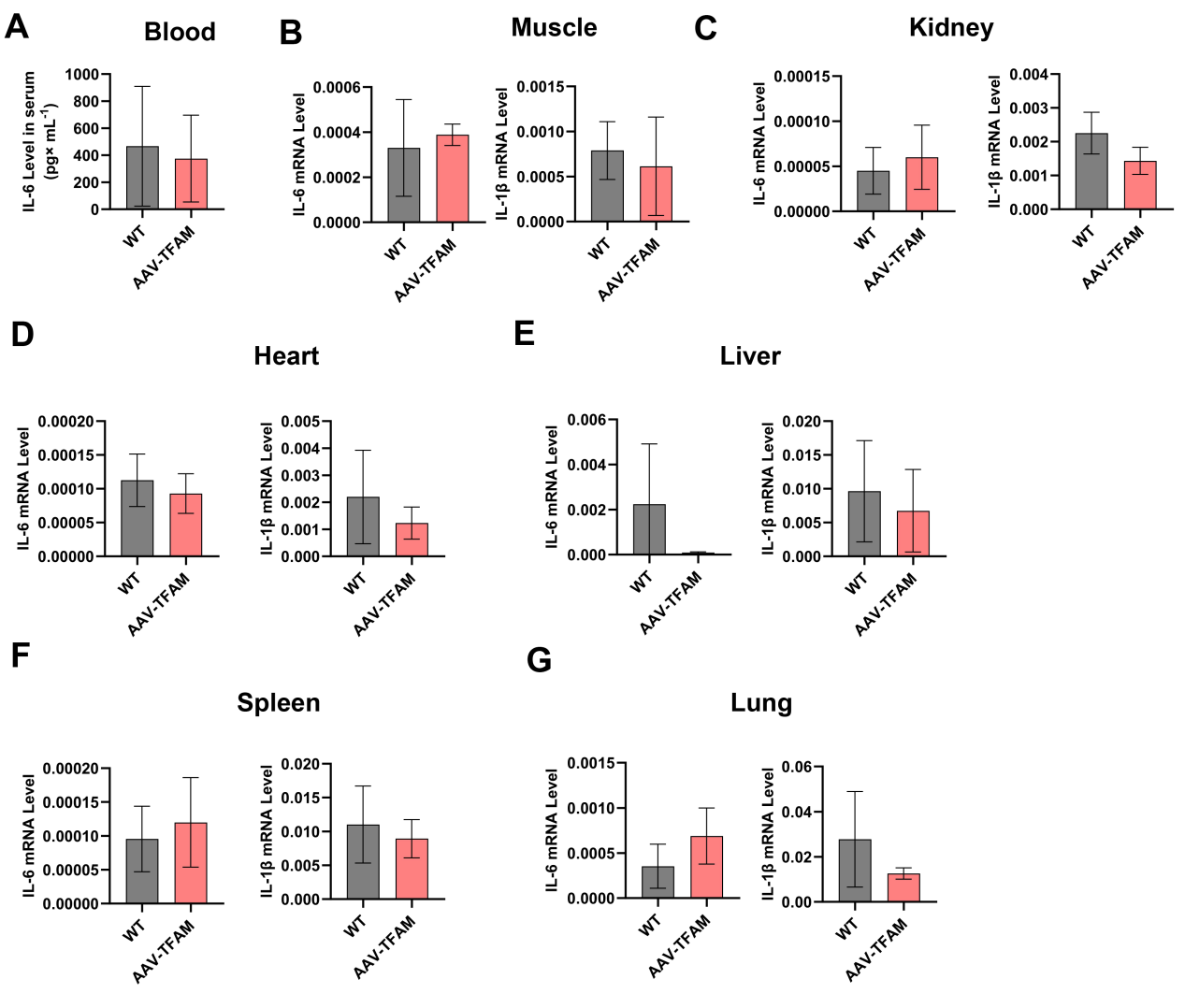
**

**Figure S17 *In vivo* immunogenicity evaluation of AAV-TFAM vector.** (A) IL-6 level in the serum of the mice (n= 6 mice). (B-G) qPCR analysis of IL-6 and IL-1β gene expression in organs (muscle, kidney, heart, liver, spleen and lung, n = 6 mice).

**Table S1. Real-time PCR primers used in the study**

| **Gene** | **Sequence 5'-3'** | **Species** |
| --- | --- | --- |
| tfam | CACCCAGATGCAAAACTTTCAG | Mouse |
|  | CTGCTCTTTATACTTGCTCACAG |  |
| acca | CCCAGAGATGTTTCGGCAGTCAC |  |
|  | GTCAGGATGTCGGAAGGCAAAGG |  |
| acly | ACAGTCAGGCGGAGGAGTTCTAC |  |
|  | TTCTGGGCTTTGGCATCCACATC |  |
| fasn | GGATATTGTCGCTCTGAGGCTGTTG |  |
|  | TGCTCCTTGCTGCCATCTGTATTG |  |
| hgf | GGATTCGCAGTACCCTCACAAGC |  |
|  | AGCAGTAGCCAACTCGGATGTTTG |  |
| fgf21 | CTGGGGGTCTACCAAGCATA |  |
|  | CACCCAGGATTTGAATGACC |  |
| vegfa | CTCACCAAAGCCAGCACATA |  |
|  | CCTTTCCCTTTCCTCGAACT |  |
| rps18 | TTCGCCATCACTGCCATTAAGGG |  |
|  | ATCACTCGCTCCACCTCATCCTC |  |
| tnf-α | ACGGCATGGATCTCAAAGAC |  |
|  | AGATAGCAAATCGGCTGACG |  |
| il-6 | AAATGATGGATGCTACCAAACT |  |
|  | CTCTGGCTTTGTCTTTCTTGTT |  |
| fn | GCAAACCTATAGCTGAGAAGTG |  |
|  | CAAGTACAGTCCACCATCATC |  |
| gapdh | CAGATCCACAACGGATATATTGGG |  |
|  | CATGACAACTTTGGCATTGTGG |  |
| il-1β | GTCCTGTGTAATGAAAGACGGC |  |
|  | CTGCTTGTGAGGTGCTGATGTA |  |

| **Gene** | **Sequence 5'-3'** | **Species** |
| --- | --- | --- |
| smpd3 | GCGAATTGGTGGCGAGGAAGG | Mouse |
|  | GCTGATTGTGGTTGGGTGTCTGG |  |
| pdcd6ip | GTTCCTCCTGCTTCTGCTGCTG |  |
|  | ATGGTGGTCCCTGAGCCTGTG |  |
| snx4 | TGGAGGCGGTACAGTGAGTTCG |  |
|  | CTGGGAGAGGTGGCACAACAAC |  |
| cd63 | GCCATTGGTGTAGCGGTTCAGG |  |
|  | GCAACAGCGAGCCAGCAGTAG |  |
| bax | TGGAGATGAACTGGACAGCA |  |
|  | TGAAGTTGCCATCAGCAAAC |  |

**References:**

1. Liu, S. et al. Improving the Circulation Time and Renal Therapeutic Potency of Extracellular Vesicles Using an Endogenous Ligand Binding Strategy. *J. Control. Release* **352**, 1009-1023 (2022).
